# Indication for selfing in geographically separated populations and evidence for Pleistocene survival within the Alps: the case of *Cylindrus obtusus* (Pulmonata: Helicidae)

**DOI:** 10.1186/s12862-017-0977-0

**Published:** 2017-06-13

**Authors:** Luise Kruckenhauser, Elisabeth Haring, Barbara Tautscher, Luis Cadahía, Laura Zopp, Michael Duda, Josef Harl, Helmut Sattmann

**Affiliations:** 1 Natural History Museum Vienna, Central Research Laboratories, Burgring 7, 1010 Vienna, Austria; 2 Department of Integrative Zoology, University of Vienna, Althanstraße 14, 1090 Vienna, Austria; 3 Third Zoological Department, Natural History Museum Vienna, Burgring 7, 1010 Vienna, Austria

**Keywords:** *Cylindrus obtusus*, Endemic species, Glacial refugia, Selfing, Gastropoda, Phylogeography, Microsatellites, COI

## Abstract

**Background:**

*Cylindrus obtusus* is one of the most prominent endemic snail species of the Eastern Alps. It is restricted to alpine meadows and calcareous rocky habitats above 1500 m. Peculiar intraspecific differences have been observed in its genital tract in the eastern populations the two mucus glands associated with the love dart sac are highly variable, while almost no variation was observed in the western populations. This raises the question whether the mode and success of reproduction of the respective populations are different. To find out whether these anatomical differences reflect genetic differentiation, which might be an indication for distinct glacial refugia, we investigated a 650 bp fragment of the mitochondrial *cytochrome oxidase subunit 1* gene (*COI*) (280 individuals) and 9 microsatellite loci from samples (487 individuals from 29 populations) covering the whole distribution range of the species.

**Results:**

The *COI* sequences show a geographic differentiation between eastern, central and western populations. The westernmost localities, which were covered under ice sheets during glacial periods, are characterized by extreme low variability. Overall genetic distances among all individuals are small (max. 1.7% *p*-distance). The microsatellite analysis reveals a high differentiation between populations, implying restriction of gene flow. The highest genetic variability was found in the central populations. Remarkably, nearly all individuals from the eastern populations, which are more variable in their genital morphology, are homozygous in all microsatellite loci, although different alleles were found within populations.

**Conclusions:**

The most peculiar outcome of the study is the strong evidence for selfing in *C. obtusus* as indicated by the microsatellite data in the easternmost populations. This finding is supported by the deformation of the mucus glands in the same populations. Since mucus glands play an important role in sexual reproduction, it seems plausible that in selfing organisms these structures are reduced. The phylogeographic structure revealed by *COI* sequences implies that the species has survived the ice ages within the Calcareous Alps. The small genetic distances among all individuals (max. 1.7%) suggest that *C. obtusus* has experienced severe bottlenecks in the past.

**Electronic supplementary material:**

The online version of this article (doi:10.1186/s12862-017-0977-0) contains supplementary material, which is available to authorized users.

## Background

*Cylindrus obtusus* (Draparnaud, 1805) [1] is a pulmonate land snail species endemic to the Eastern Alps of Austria where it is restricted to primarily forest-free sites [2], at elevations between 1600 and 2500 m [3]. The geographical distribution has been listed in detail by several authors (e.g. [4, 5]) and covers the easternmost part of the Northern Calcareous Alps and some limestone islands within the Central Alps [4]. Ecologically, *C. obtusus* is restricted to alpine meadows and calcareous rocky habitats from the subalpine ecotone upwards [2, 5–7], hence its distribution is rather scattered. Dispersal potential and reproduction rate seem to be rather low, whilst populations can be quite dense and life span is assumed to exceed 10 years [3]. Within the subfamily Ariantinae *C. obtusus* is peculiar because of its aberrant cylindrical white shell, the other representatives of the Ariantinae display globular or more or less depressed shells [8]. The presumed sister group relationship of *Cylindrus* Fitzinger, 1833 and *Arianta* Leach, 1831, the latter being a genus widespread in Europe and also common in the Alpine region, is supported by anatomical features and molecular genetic data [9–12]. Boettger [13] and Schileyko [14] argue for a rather young origin due to recent mutation, but according to molecular genetic data the split between the two morphologically distinct genera is probably quite old [11, 12]. In general, the distribution pattern of *C. obtusus* and colonization of formerly glaciated areas of the Alps is not yet well understood and discussed controversially [12–16]. The fossil record does not provide much information because conditions for fossilization are unfavourable in high mountain areas and dating is problematic [17]. Due to its outstanding position within the Ariantinae in terms of shell morphological differentiation and geographical restriction to high mountain regions, *C. obtusus* is one of the most prominent endemics of the Eastern Alps.

Concerning the intraspecific variation, studies on shell morphology have evidenced some geographical differences in shell size in different populations [15, 18], whereas anatomical studies revealed surprising differences in characters of the genital tract [19–21]. These concern particularly the size variation of the two mucus glands associated with the stylophore, the organ in which the love darts are produced and stored (also called dart sac). This size variation was first reported by Schileyko [20, 22], who found that the length of the mucus glands varies significantly in the easternmost populations, while no significant variation was observed in the western ones. The size differences were not measured quantitatively and thus the data could not be analyzed statistically. A comprehensive analysis of shell morphology, genital anatomy and histology on material covering the whole distribution range was performed recently by Zopp et al. [21], who confirmed and quantified the differences in anatomical traits and shell size between eastern and western populations. On the one hand the remarkable geographical differentiation might reflect a phylogenetic split; on the other hand the variation observed in the genital anatomy might have also influenced the mode and success of reproduction of the respective populations. Several questions arose from these findings: (1) Are the morphological and anatomical differentiations the result of genetic divergence between the eastern and western populations? (2) Do eastern and western populations differ in their genetic variability or population structure? (3) Is the scattered distribution of populations reflected by genetic isolation? (4) Do genetic data support the hypothesis that *C. obtusus* survived the ice age within the Calcareous Alps as suggested by [2].

In the present study comprehensive molecular genetic analyses covering the whole distribution range of *C. obtusus* were performed in order to address these questions. We present results from a DNA sequence analysis of sections of the mitochondrial (mt) cytochrome oxidase subunit I (*COI*) gene as well as a population genetic analysis based on nine microsatellite loci.

## Results

### *COI* sequence variation

**Fig. 1:**
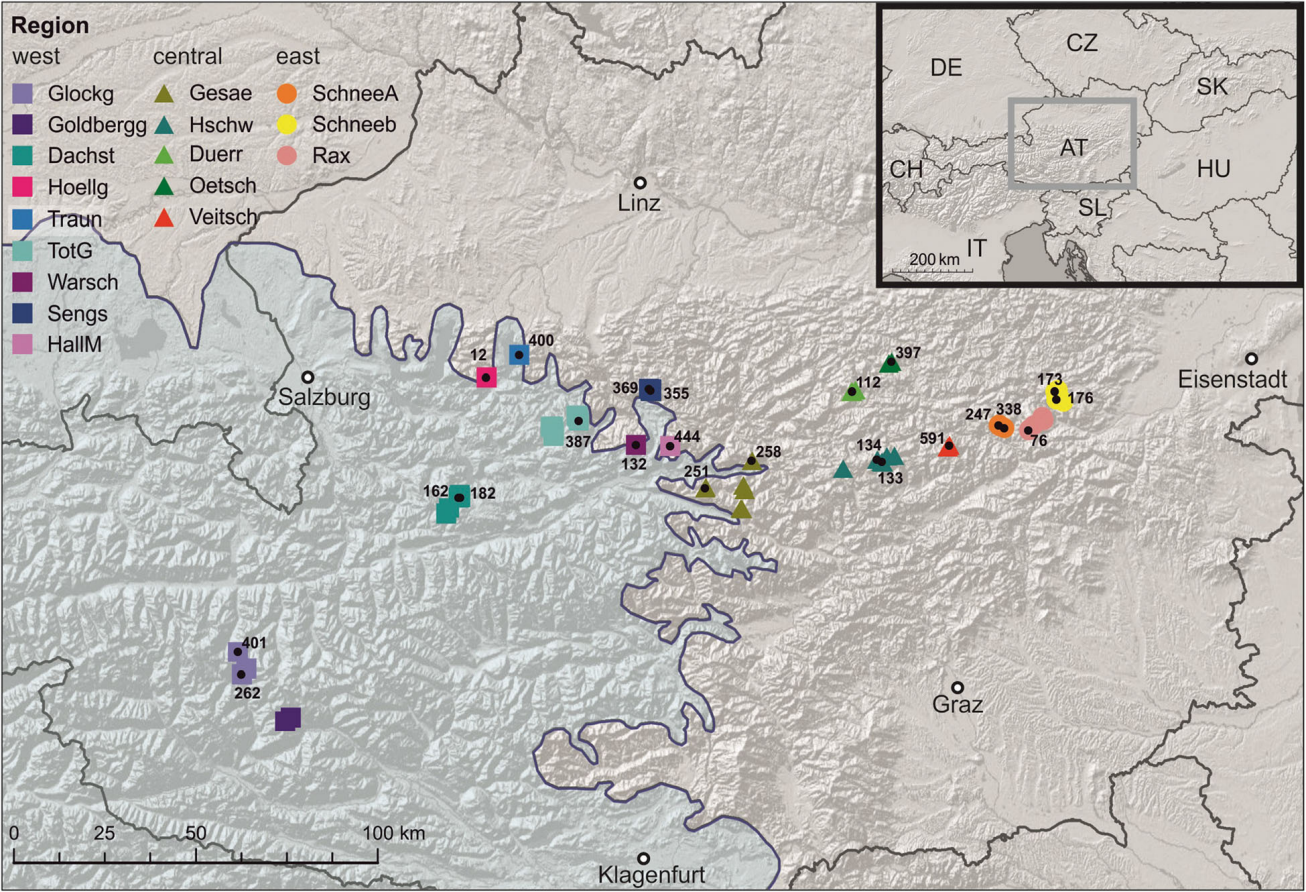
Sampling localities of *C. obtusus* covering the whole distribution range of the species. In several regions sampling sites were too dense to be depicted as separate circles (for details of sampling sites see Additional file 6). Localities used in microsatellite analysis are indicated by *black* dots. Colour codes refer to the geographic regions, numbers to the sampling sites. The *dashed blue* line indicates the maximum extent of glaciers (35–19 ka ago) during the Wormian glacial (van Husen 1994). The abbreviations of the geographic regions are as following: Glockg (Glocknergruppe), Goldbergg (Goldberggruppe), Dachst (Dachstein), Hoellg (Höllengebirge), Traun (Traunstein), TotG (Totes Gebirge), Warsch (Warscheneck), Sengs (Sengsengebirge), HallM (Haller Mauern), Gesae (Gesäuse), Hschw (Hochschwab), Duerr (Dürrenstein), Oetsch (Ötscher), Veitsch (Veitsch), SchneeA (Schneealpe), Rax (Rax), Schneeberg (Schneeberg)

**Fig. 2:**
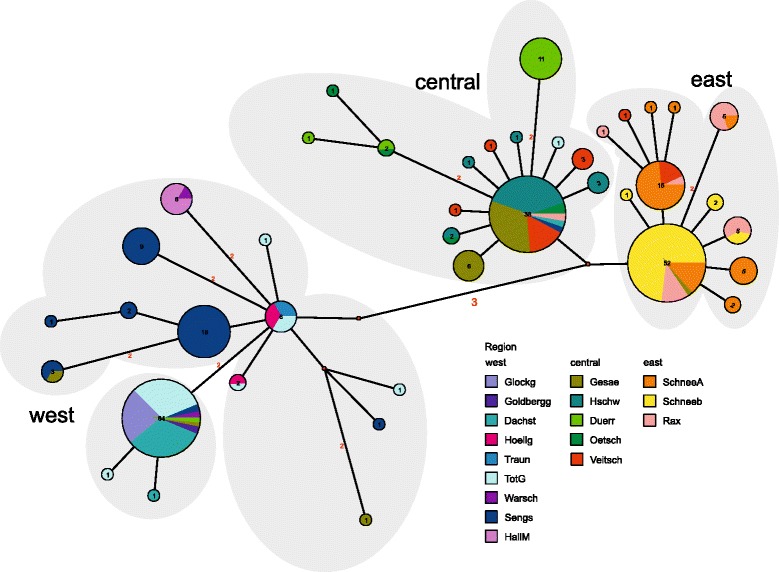
Median-Joining network of *COI* haplotypes. Colours of the haplogroups refer to the geographic regions defined in Fig. 1. The grouping according to spatial clustering as deduced by BAPS is indicated by *grey* shades

In the set of 280 individuals from 94 localities (Fig. 1) analyzed for a section of the mt *COI*, 41 haplotypes were found. Over the whole distribution range genetic variation is rather low with a maximum of 1.7% (uncorrected *p*-distances). The Median Joining network (Fig. 2) shows that the haplotypes follow a geographic pattern. There are three main haplogroups, one of them mainly consisting of western populations (from the Glocknergruppe to the Haller Mauern; 38 localities), one comprising almost exclusively samples of the easternmost populations (eastern mountain ranges from Veitsch to Schneeberg; 37 localities) and a central one consisting mainly of populations from a central area (Veitsch to Gesäuse; 19 localities). Populations from one region (Veitsch) possess haplotypes from both the eastern (4) and the central (11) haplogroup. In each of the haplogroups there are also few individuals from regions which are found also in the other two haplogroups. E.g., four individuals from Gesäuse (out of 22) possess haplotypes from the eastern (1) or western (3) haplogroup, respectively. In each of the three haplogroups there is one frequent and widely distributed haplotype, e.g., one in the western group shared by 64 individuals covering a geographic range from Glocknergruppe to Dürrenstein. Interestingly, all individuals from Glocknergruppe and Goldberggruppe share this most frequent haplotype. In order to illustrate the distribution of haplotypes in detail and to depict the position of single individuals a NJ tree is presented in Additional file 1.

In order to evaluate the differentiation into three haplogroups we performed a PCA analysis and spatial clustering as implemented in BAPS. The PCA clearly separates the three haplogroups (western, central and eastern group) in different clusters, with the first two components explaining 73.9% of the variance (see Additional file 2) The spatial clustering in BAPS found an optimum at four genetic groups (see Fig. 2): the eastern and central group was the same as in the PCA, the western group was split in two groups: one consisting of individuals from Höllengebirge, Traunstein, Warscheneck, Haller Mauern and Sengsengebirge the other of individuals from the Goldberggruppe, Glocknergruppe, and Dachstein, the individuals from Totes Gebirge are distributed in both groups. Single individuals (as already seen in the network) are assigned to groups that are otherwise geographically more distant. The second of the two western groups consists of individuals of a single haplotype (and two singletons with one substitution to the main haplotype) which are from the westernmost localities, all of which have been covered under the icesheet during the Last Glacial Maximum. The haplogroups are quite closely related (Table 1). The main haplotypes of the eastern and the central group are separated by only two substitutions, while the western group is separated by at least five substitutions (mean *p*-distance 1.0%). Nucleotide diversity within groups is generally quite low, especially in the eastern haplogroup, and the same is the case for the *p*-distances between groups. On the other hand haplotype diversity is similar in all three groups. Distances between haplogroups as well as nucleotide and haplotype diversities are summarised in Table 1.

**Table 1:** Mean and maximum p-distances within and among clades; nucleotide and haplotype diversity of clades

	East	Central	West
East	0.1 (0.5)	--	--
Central	0.5 (1.1)	0.2 (0.6)	--
West	1.0 (1.5)	1.0 (1.7)	0.3 (0.8)
N	91	72	117
H	12	14	15
h	0.6427 +/− 0.0511	0.6944 +/− 0.0542	0.6699 +/− 0.0437
pi	0.001439 +/− 0.001101	0.001960 +/− 0.001376	0.003234 +/− 0.002006

Upper part: Mean and maximum (in parentheses) *p*-distances in %. Lower part: *N* number of individuals, *H* number of haplotypes, *h* haplotype diversity, *pi* nucleotide diversity, behind the slash: standard deviation

The comparison of diversity values between geographic regions (Additional file 3) reveals low values for populations within the formerly fully glaciated region (Glocknergruppe, Goldberggruppe, and Dachstein) and high values in several regions at the periphery of the glaciated region (from Höllengebirge to Gesäuse; up to 0.7). Populations of more eastern regions have mostly moderate to high haplotype diversities with the highest value found in Ötscher (0.9). The overall lowest diversity was found in the easternmost mountain range Schneeberg (0.25) despite the fact that this region is represented by the largest number of samples (44 individuals, 17 populations).

### Microsatellite variation

#### Genetic variability within localities

In general the microsatellite variation is high, with 10 to 28 different alleles per locus across all localities. The AMOVA showed that most of the variation is explained within population (54.09% vs. 45.91% among populations). Variability within populations differed remarkably between localities being generally low in the westernmost and easternmost populations (including Veitsch_591) and higher in the central populations (from Traun_400 to Oetsch_397). With respect to the private alleles the population Duerr_112 shows a value considerable higher than all the other localities (0.61 vs. the next smaller value of 0.26; see Table 2 and Additional file 4).

**Table 2:** Population genetic parameters of localities

Population	N	A	Ar	pA	HO	HE	Fis	p	Ld	Lp%	s
Glockg_401	28	3.88	2.50	0.07	0.44	0.43	−0.04	0.204		100	0.00
Glockg_262	20	3.00	1.67	0.04	0.20	0.21	0.04	0.771		88	0.00
Dachst_162	29	4.00	2.47	0.00	0.31	0.33	0.08	0.597		78	0.01
Dachst_182	25	2.78	2.09	0.04	0.24	0.29	0.15	0.140		67	0.00
Hoellg_12	10	3.13	2.92	0.03	0.39	0.42	0.09	0.890		88	0.55
Traun_400	8	4.88	3.61	0.03	0.58	0.67	0.15	0.273		88	0.00
TotG_387	27	7.75	4.21	0.22	0.66	0.71	0.07	0.614		100	0.04
Warsch_132	30	7.11	4.14	0.30	0.58	0.66	0.12	High. sign.	CO16, CO30	100	0.00
Sengs_369	27	6.22	3.83	0.11	0.59	0.64	0.07	0.218		100	0.00
Sengs_355	19	7.00	4.73	0.12	0.71	0.74	0.04	0.800		100	0.00
HallM_444	6	3.38	3.08	0.26	0.38	0.51	0.28	0.055		88	0.28
Gesae_251	24	4.67	2.99	0.21	0.59	0.55	−0.08	0.000	CO16, CO50	100	0.00
Gesae_258	19	8.22	4.92	0.23	0.72	0.77	0.06	0.049	CO30	100	0.00
Duerr_112	29	6.44	3.67	0.61	0.60	0.62	0.03	0.462		100	0.08
Hschw_134	26	5.67	3.44	0.19	0.59	0.63	0.06	0.000	CO21	100	0.07
HSchw_133	10	3.56	2.61	0.14	0.38	0.45	0.16	0.493		100	0.00
Oetsch_397	17	6.67	4.48	0.18	0.72	0.75	0.05	0.900		100	0.08
Veitsch_591	26	2.50	1.98	0.06	**0.05**	**0.27**	**0.83**	**0.000**	**all**	75	**0.82**
SchneeA_247	14	1.88	1.69	0.00	**0.00**	**0.22**	**1.00**	**0.000**	**all**	75	**no he**
SchneeA_338	30	1.50	1.36	0.01	**0.00**	**0.09**	**1.00**	**High. sign.**	**all**	38	**no he**
Rax_76	20	2.75	2.02	0.00	**0.03**	**0.29**	**0.92**	**High. sign.**	**all**	100	**0.93**
Schneeb_173	23	3.50	3.04	0.10	**0.02**	**0.55**	**0.97**	**High. sign.**	**all**	100	**0.96**
Schneeb_176	20	3.75	2.93	0.00	**0.12**	**0.51**	**0.77**	**High. sign.**	**all**	100	**0.85**

Number of individuals analyzed (N); mean number of alleles per locus (A); allelic richness, sample size (g) = 10, rarefaction approach (Ar); number of private alleles, sample size (g) = 12 rarefaction approach (pA); observed (HO) and expected (HE) heterozygosity; inbreeding coefficient (Fis); probability of departures from Hardy–Weinberg equilibrium (p); loci, that deviated from the HW-equilibrium (Ld); percentage of polymorphic loci (Lp%); estimated selfing rate (s). Those parameters that indicate selfing in the eastern populations are printed in bold

Several population parameters separate the easternmost localities (Veitsch_591 to Schneeb_176), from all the others. These populations also belong to the eastern mitochondrial clade. The only exception is Veitsch, which has haplotypes belonging to the central as well as the eastern haplogroup. The only sequenced individual of the location Veitsch_591 (which we used for microsatellite analysis) has an eastern haplotype. These parameters include the low observed heterozygosity (0–0.12), very high Fis values (0.83–1) and high *p*-values for the deviation from the Hardy-Weinberg equilibrium for all loci. Also the estimated selfing rate per population is much higher in the eastern populations (all above 0.82). For the two populations SchneeA_247 and SchneeA_338 selfing rate even could not be estimated, due to the lack of any heterozygotes (although several alleles were present).

#### Differentiation of the localities

**Fig. 3:**
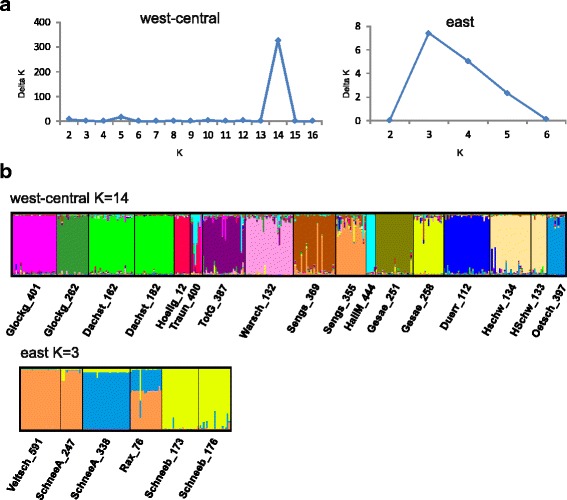
Bayesian cluster analysis for *C. obtusus* performed using STRUCTURE. **a** Calculation of delta K for all tested K. **b** Each column along the x axis represents one individual

The PCA of the microsatellite data (approx. 10% of the variance is explained by the first two components; see Additional file 2) show the localities in clusters which are partly overlapping and arranged in a continuous west to east pattern, with only the populations from Dachstein being more separated. In the PCA of the microsatellite data no grouping congruent to the mitochondrial haplogroups was found. This is supported by the AMOVA when grouping is performed according to the mt haplogroups (only Veitsch assigned to the eastern group). The variation among groups is rather low (9.53%) compared to those among populations within groups (37.98%) and within populations (52.49%). The isolation by distance calculation showed a strong correlation between the genetic and the geographic distances (Z = 10,679,583.3384, *r* = 0.4574, *p* < 0.0010). This was even stronger when we used only the western and central (excluding Veitsch) populations (Z = 4,269,718.0793, *r* = 0.5512, *p* < 0.0010), while the eastern populations (including Veitsch) showed no significant correlation (Z = 130,867.1952, *r* = −0.1158, *p* = 0.6210; see Additional file 5). For the STRUCTURE analysis it is assumed that within populations, the loci are at Hardy-Weinberg and linkage equilibrium [23]. This is not the case for the eastern localities including Veitsch (see Table 2). To ensure that the analyses of the western and central localities are not influenced by the violation of this requirement, they were treated separately from the eastern localities in the STRUCTURE analysis. For the 17 west/central localities the highest probability, mean LnP (K), was found for K = 14, this was also the number of K with the highest Delta K. The following localities joined in single groups in the STRUCTURE analysis: Dachst_162/Dachst_182 and Hochschw_134/Hochschw_133. The locality Traun_400 (from which only eight individuals were analyzed) appeared to be an admixture of Hoellg_12 and HallM_444 (Fig. 3b). For the six eastern localities the STRUCTURE results were ambiguous concerning the number of groups. The highest DeltaK was for K = 3, while the highest mean LnP (K) was found for K = 7. For K = 6, which is the number of localities, all of them represent distinct populations, indicating some geographic differentiation. The following localities were combined in single populations by STRUCTURE: Veitsch_591/SchneeA_247 and Schneeb_173/Schneeb_176. The locality Rax_76 showed to be an admixture of SchneeA_338 and Veitsch_591/SchneeA_247 (Fig. 3b). However, these results have to be treated with caution as structure itself assigns individuals to populations assuming that these are under Hardy – Weinberg equilibrium, which is not true for any of the eastern localities.

## Discussion

### Genetic variability and phylogeography

The overall genetic diversity within *C. obtusus* as observed in the section of the mt *COI* gene, is remarkably low (max 1.7%) compared to other Alpine land snail species with similar habitats, e.g., *Pyramidula pusilla* (Vallot, 1801) [24] or *Orcula dolium* (Draparnaud, 1801) [25]. In the latter species three strongly differentiated *COI* clades were found in the area, which were separated by distances of up to 15.8%. Another species occurring in a similar habitat is *Arianta arbustorum*, a member of the genus closest related to *Cylindrus*, which is separated by a genetic p-distance of 20% from *C. obtusus* [12], has a high intraspecific variability (of more than 12.5% distance in the *COI*; [26]. However, all those species have quite large distribution ranges and broader ecological niches compared to *C. obtusus*, which might explain why they were able to maintain high diversity over a long period. Yet, in *Trochulus oreinos* (A. J. Wagner, 1915), another species endemic to the Eastern Alps with similar but more restricted habitat needs and an even smaller distribution range, which is widely overlapping with that of *C. obtusus* [2], high intraspecific genetic differentiation has been observed*.* Its two subspecies, the eastern *T. o. oreinos* and the western *T. o. scheerpeltzi* (Mikula, 1954) are separated by 13.7% average p-distance in the *COI* [27]. The variation in the *COI* within each of the two subspecies is similar to that found in *C. obtusus* (*T. o. oreinos*: 1.4*%, T. o. scheerpeltzi*: 0.9%). Both *C. obtusus* and *T. oreinos* are species adapted to limestone mountain regions above 1500 m asl [2]. Thus, they probably could not shift their distribution ranges in glacial periods to adjacent lower regions as easily as other species, e.g., *A. arbustorum* and *O. dolium*, which also inhabit lowland areas surrounding the Alps. But even the climate during warmer periods, when treelines moved up to higher elevations, might have contributed to fragmentation of habitats and subsequently to loss of diversity as well. In summary, we assume that *C. obtusus* has experienced severe bottlenecks in the past.

The differentiation in three closely related haplogroups indicates that the phylogeographic pattern as displayed in *C. obtusus* has been shaped during several glacial periods and that at least three Late Pleistocene refugia existed for this species (west, central and east). There is a general scarcity of terrestrial gastropod fossils of the Miocene to the Pleistocene in Alpine mountain regions. Also from *C. obtusus* only some rare and mostly Holocene fossils within the extant distribution range in the Eastern Alps are documented and no scientific datings for these are available ([17]; for detailed discussion see [12]). Therefore, a reliable calibration of a molecular clock and thus dating of the splits of the three lineages is not possible based on data presently available. Yet, a rough comparison with the molecular clock analysis of species in the genus *Orcula* Held, 1837 [28] implies that the lineages probably split not earlier than in the middle Pleistocene. Thus the colonization of the current distribution range of *C. obtusus* most likely started within that time frame.

Especially the western and the eastern group show a star-like haplotype pattern in the network, which indicates bottlenecks during the last glaciation, in which the population size of these groups might have been drastically reduced. From the western group at least the localities of Glocknergruppe, Goldberggruppe and Dachstein were covered by glaciers during the Last Glacial Maximum (LGM) 30–18 kya [29] (Fig. 1). This is well reflected in the mitochondrial data, because almost all individuals share the same haplotype (only one substitution was found in a single individual), which also occurs frequently in Totes Gebirge. This location was also covered by ice during the LGM, but harbours a higher genetic diversity. This can be explained by survival at the periphery of the ice sheet, in so called peripheral nunataks, as it has proposed for several other species [26, 30, 31].

The occurrence in the formerly glaciated Alpine regions indicates that colonization might have happened rather fast. Also the geographic pattern in the mitochondrial network is not perfect as exemplified, e.g., by the individuals from Gesäuse (geographically representing the central group) possessing haplotypes of the eastern or western haplogroups, respectively. These results imply that *C. obtusus* might be capable of sporadic long distance migration, which is possibly driven by passive transport (e.g., by birds or other vertebrates) as it has been shown for other snail species [32, 33], but also transport with strong winds. However, considering the still strong pattern of geographic distribution, long distance migration does not seem to be a frequent event.

While the mitochondrial data show low within species variability, the variation of the microsatellites is high, as expected for a neutral markers. The microsatellites show a high structuring of the sampled locations, as it can be expected for taxa with generally low migration ability. In the structure analysis of the western and the central group only two of the 17 locations were merged into one cluster with the neighbouring locations. In both cases the sampling localities were quite close to each other (500 and 1500 m) and hence the merging in the same population is not surprising. However, also the two localities from Sengsengebirge are only 500 m away from each other and separated as two clusters by STRUCTURE, indicating that population size in *C. obtusus* might differ among regions and mountain stocks. Concerning the within population variability as calculated from the microsatellite data, the lowest values were found at the western and the eastern margin of the distribution area, which is in accordance with the mitochondrial data.

### Selfing in the eastern populations

All populations east of Ötscher show a very peculiar population structure, as they have a high excess of homozygote individuals, which is significant for all loci in these six populations (133 individuals). All these populations had different alleles (mean number per locus ranging from 1.88 to 3.75) but nearly no individuals with heterozygote loci; this resulted in observed heterozygosities which were in magnitudes lower than the observed heterozygosities. As a consequence, in these populations the deviation from the Hardy-Weinberg equilibrium was significant in all loci analysed. In theory the violation of the Hardy-Weinberg equilibrium can have several reasons: finite population size, selection, genetic drift and high geneflow are not explanatory for the observed pattern in the eastern populations of *C. obtusus,* because these populations are large in number of individuals, all analysed loci are affected and also the data do not point towards recent population mixture. An independent deficiency of heterozygotes in several populations and in all the loci analysed cannot be explained by genetic drift or sampling effect (14 to 30 individuals were analysed per population). Hence, the remaining reasons for deviation from Hardy –Weinberg equilibrium have to be taken into consideration: non-random mating due to population sub-structuring and inbreeding or selfing. Concerning the population sub-structuring, we applied the same sampling regime for the eastern populations as for all the other regions and there was no indication for different population structure or very distinct habitat conditions compared to the other (more westerly) regions, therefore this is not a reasonable explanation.

It is difficult to distinguish between inbreeding and selfing on the basis of the population genetic data alone. In order to elucidate this issue, we attempted to conduct breeding experiments with *C. obtusus* and kept individuals for more than 4 years, but without any breeding success. Another approach to distinguish inbreeding from selfing would be a fine-scale population screening, which we plan in a further investigation. Interestingly, the variability in genital anatomy of *C. obtusus* (in particular the dart sac and the mucus glands) proved to be different in eastern populations compared to western and central populations [20]. In contrast to the western and central populations, which are quite homogenous in their genital anatomy, the eastern populations exhibit a high variability in the length of the mucus glands, sometimes being even disproportionate within one individual, which is quite uncommon in helicid snails [34, 35]. These peculiarities were analyzed in detail in a large sample by Zopp et al. [21]. Among others, [36] could show with injection experiments that the mucus from the mucus glands, that covers the love dart before injection, is the reason for the increased probability for paternity of the dart shooting partner. This feature would, of course, not be necessary in a selfing organism. Hence the co-occurrence of the deformation of the mucus glands and the high excess of homozygosity supports the hypothesis of a high degree of selfing in the eastern populations. This finding is also supported by the calculated selfing rates between 82% and 96% in the eastern populations.

Selfing is a known phenomenon in various hermaphrodite pulmonates, most prominent in basommatophoran freshwater snail species, where delayed selfing can be observed during colonization when mating partners are absent [37–39]. The evidence in stylommatophoran gastropods is more scarce (e.g., predominantly selfing is found in *Deroceras laeve* and *Balea perversa* [40] as well as *Arion sylvaticus* and *A. circumscriptum* [41]). It was also found in *A. arbustorum*, the closest relative *of C. obtusus*, but in this species selfing was observed only in laboratory experiments at very low rates and the offspring displayed inbreeding depression [42]. Hence, this phenomenon, which is believed to have evolved to assure reproduction even in the absence of a partner, is counteracted by inbreeding depression [43].

While in several molluscs it is discussed how a mixed breeding model can be maintained with intermediate selfing rates (eg. [38, 43]), in *C. obtusus* there seems to be a clear geographic pattern. The western and central populations have quite low estimated selfing rates; in the eastern populations these estimates were so high, that predominantly, if not exclusively, selfing can be assumed. The geographic differences in genital anatomy cannot be reasonably explained by ecological and population density factors, because at least today there seems to be no big difference in habitat parameters and population densities [2]. The sequence data indicate that the eastern populations re-established after a severe bottleneck, probably during or even before the LGM. A possible scenario would be that, due to drift effects, the altered reproduction mode might have become fixed.

Cryptic species are a known phenomenon especially in gastropods, which might lead to underestimation of animal diversity [44]. The overlapping and continuous west to east pattern in the PCA of the microsatellite data, the significance of the isolation by distance as well as the shared haplotypes between the geographic regions (6 individuals between west and central) suggests that *C. obtusus* represents a single species and that there are no cryptic species hidden in this taxon. However, the altered mode of reproduction in the eastern populations can be considered as a factor that could foster isolation and thus future speciation.

## Conclusions

The phylogeographic results based on *COI* sequences revealed a geographic differentiation between eastern, central and western populations of *C. obtusus*, suggesting survival in several refugia during the last glacial periods which were probably accompanied by severe bottlenecks.

In the current study we discovered an exceptional example of different reproduction modes in geographically separated populations. On the basis of microsatellite data we conclude that the easternmost populations of this hermaphroditic land snail species reproduce predominately by selfing. This finding is supported by the deformation of the mucus glands in the same populations. Mucus of these glands covers the love dart in the course of normal sexual reproduction in hermaphroditic pulmonate land snails and is the reason for increased probability of paternity for the dart shooting partner. The more western populations have homogenously developed mucus glands and the population genetic parameters show no signs for selfing. Our phylogeographic data indicate that these eastern populations survived the last glacial maximum in a separate refugium. Thus, we assume that, probably due to drift effects during glacial bottlenecks and postglacial expansion in the eastern habitats, an altered reproduction mode might have become predominant. It remains to be investigated whether interbreeding between the two forms still may occur.

## Methods

### Samples and localities

The samples of *C. obtusus* investigated in the present study were collected in the years 1997 to 2010 and cover the whole distribution range. Additional file 6 summarizes information on the collection sites (geographic region, latitude and longitude) and Fig. 1 shows their geographic location. Positions of collection sites were determined by GPS according to the World Geodetic System 1984 (WGS84) or localized with Geogrid-Viewer 3.1. For DNA sequence analysis 280 individuals from 94 localities were used (Additional file 6). For the microsatellite analysis a set of 23 populations was selected each of which consisted of up to 30 specimens (Additional file 6). With two exceptions those populations comprise at least 10 individuals, two populations contain only six (HallM_444) or eight (Traun_400) individuals, respectively (Table 2). As the latter two are important from a phylogeographic point of view, they were included in spite of the low sample size. Altogether 487 individuals were included in the microsatellite study. In general, we used the individuals sequenced also for the microsatellite analysis, but for some localities the individuals were only used for sequencing, therefore a total of 692 individuals was included into the study. Specimens were treated following the protocol of Kruckenhauser et al. [45] and stored in 80% ethanol. All voucher specimens were deposited in the molluscan collection of the 3rd Zoological Department of the Natural History Museum Vienna (voucher numbers see Additional file 7). From each individual a tiny piece of the foot tissue was taken for DNA extraction. Remaining tissue and DNA are stored in the DNA and tissue collection of the NHMW.

### Molecular genetic analysis

DNA was extracted with the First-DNA all-tissue Kit (GEN-IAL), following the manufacturer’s protocol and a final elution volume of 100 μl. A section of the mt *COI* was amplified with the primers CO1alb_fw (5′-CCA CTA ACC ACA AAG ATA TTG GGA C-’) and CO1Cyobt1_rv (5′-ATT AGA ATA TAC ACT TCC GGA TGG Cc-3′). The resulting sequence (without primers) was 662 bp in length. PCRs were performed on a Master Gradient thermocycler (Eppendorf) in 25 μl with 1–3 μl template DNA, 0.5 unit Taq DNA polymerase (Roche), 0.5 μM of each primer and 0.2 mM of each dNTP (Roche). Each PCR comprised 35 reaction cycles with an annealing temperature of 50 °C. Control reactions were carried out for both DNA extractions and PCR amplifications. PCR products were purified using the QIAquick PCR Purification kit (Qiagen) and analyzed by direct sequencing (both directions). Sequencing was performed at LGC Genomics (Berlin, Germany) using the original PCR primers.

Microsatellites used in the present study were isolated and tested by Arthofer et al. [46]. The following nine primers were used: multiplexed loci: (1) Co16, Co44, Co50; (2) Co30, Co59; (3) Co20, Co26; non-multiplexed: Co1 and Co21. Microsatellite amplification and detection followed the procedures as described in Arthofer et al. [46]. In the individuals from some of the western localities (Glockg_401, Glockg_262, Hoellg_12, Traun_400, TotG_387, HallM_444) the locus Co16 did not amplify at all and hence was excluded. In the six easternmost localities (Veitsch_591, SchneeA_247, SchneeA_338, Rax_76, Schneeb_173, Schneeb_176) the locus Co20 showed ambiguous patterns, with up to four alleles per individuals. We concluded that in the eastern populations this locus is duplicated and discarded it for those populations. For the structure analysis both loci (Co16 and Co20) were excluded from the whole dataset.

PCR amplifications were performed in a volume of 12.5 μl using 1 μl DNA. For multiplexed loci, a QIAGEN Multiplex PCR Kit (QIAGEN, Inc.) was used with 0.2 μM of each primer and the PCR conditions described in Arthofer et al. [46]: 15 min initial denaturation and enzyme activation (95 °C), two cycles of 30 s denaturation (94 °C), 90 s annealing at locus-specific temperature, and 60 s elongation (72 °C), followed by 30 cycles of 30 s denaturation (94 °C), 90 s annealing (3 °C below initial annealing temperature) and 60 s elongation (72 °C), followed by 10 min final elongation (72 °C). For non-multiplexed loci, the PCR mixture consisted of 1.25 μl 10× PCR buffer, 0.2 mM of each dNTP, 0.32 μM of each primer, 0.5 U TopTaq DNA polymerase (QIAGEN, Inc.), and 2.5 μl 5× Q-Solution (QIAGEN, Inc.). PCR conditions were: 3 min initial denaturation (94 °C), two cycles of 30 s denaturation (94 °C), 30 s annealing at locus-specific temperature (Table 1) and 60 s elongation (72 °C), followed by 30 cycles consisting of 30 s denaturation (94 °C), 30 s annealing (3 °C below initial annealing temperature), and 60 s elongation (72 °C), followed by 10 min final elongation (72 °C). Forward primers were labelled with either 700 or 800 nm fluorescent dyes (IRD-700, IRD-800). For genotyping, fragments were separated and detected on a Li-Cor 4200 (LI-COR Biosciences) automatic sequencer. Alleles were scored using Saga Generation 2 software (Licor) and edited manually.

The sequences determined in this study are deposited in GenBank under the accession numbers: MF153098 - MF153377. Microsatellite data are stored in the Dryad Digital Repository under doi:10.5061/dryad.4gr1h.

### Data analysis

#### Sequences

Sequences were edited in BioEdit version 7.0.5.3 [47]. There were no insertions or deletions and no stop codons confirming that the sequences are derived from mtDNA rather than from numts (nuclear copies of mitochondrial sequences). Average *p*-distances were calculated using MEGA version 4 [48], which was also used to calculate a Neighbour-Joining tree [49] and to perform the non-parametric bootstrapping analysis (1000 replicates). Median-joining (MJ) networks [50] were constructed with Network 4.6.0.0 (available at www.fluxus-engineering.com), putting equal weight on each site and using the postprocessing option ‘mp calculation’. The numbers of haplotypes, haplotype and nucleotide diversities were determined with ARLEQUIN 3.11 [51]. For clustering of the sequence data we performed a PCA as implemented in PAST 3.0 [52] and the model-based Bayesian assignment as implemented in BAPS by using the geographic origin of the samples as prior information [53, 54].

#### Microsatellites

The software package ARLEQUIN 3.11 [51] was used to calculate the mean number of alleles per locus, the observed and expected heterozygosity and the AMOVA. GENEPOP version 3.1d was used to calculate the probability of deviations from Hardy–Weinberg equilibrium and their significance (P), and the inbreeding coefficient FIS, [55] was calculated with FSTAT [56]. A rarefaction approach as implemented in ADZE [57] was utilised to calculate the sample size-corrected allelic richness and the number of private alleles. A principle component analysis (PCA) was performed using the R-package ADE4 version 1.7–2 [58]. By using the scaleGen function in the R-package ADEGENET [59], which replaces the missing values with generated mean allele values it was possible to include all individuals. We calculated the geographic distances of the localities from the coordinates with the Geographic Distance Matrix Generator [60] and used this for calculation with the Isolation By Distance Web Service [61]. The estimation of the selfing rate was performed with the software RMES [62].

We used the Bayesian methodology of STRUCTURE [23] to estimate the number of populations (K), following the recommendations of [63] for reproducibility. We assumed an admixture model with correlated allele frequencies [64]. Due to their different population structure the west/central and the eastern populations were calculated independently, only the seven loci scorable in all populations were used. 20 independent runs of K = 1–17 (west/central) and K = 1–7 (east), respectively, were carried out with 500,000 Markov Chain Monte Carlo (MCMC) simulations and a burn-in of 100,000 repetitions each. The results were visualised and the Evanno method for delta K calculated with STRUCTURE HARVESTER [65]. Following Pritchard et al. [23], we used the value of K with the maximum posterior probability given by the data and Pr (X/K) to identify the most likely value for K. In addition we used delta K as described in Evanno et al. [66], the maximum second order rate of change of Pr (X/K) standardised by the standard deviation of Pr (X/K) as calculated by STRUCTURE, as an estimator for the most likely number of populations. For the STRUCTURE runs with the most likely K value, cluster assignment across replicate analyses was aligned with CLUMPP [67]. DISTRUCT [68] was used for the visualization of the aligned clusters.

## Additional files

Additional file 1: Figure S1. Neighbour joining tree of the *COI* sequences. Each individual is defined by an individual Id, the origin of geographic region, and the Id of the sampling sites. For relevant nodes, bootstrap values are indicated. (PDF 1433 kb)

Additional file 2: Figure S2. Principle component analyses (PCA) of microsatellite and sequence data. (PDF 1565 kb)

Additional file 3: Table S1. Nucleotide and haplotype diversity (standard deviation in parentheses) calculated for geographic regions. Ni = number of individuals, Nl = number of localities, H = number of haplotypes, h = haplotype diversity, pi = nucleotide diversity. (PDF 324 kb)

Additional file 4: Figure S3. Summarising in a rarefaction approach the Allelic Richness of sample size g = 10, g = 20, and g = 30 and the mean number of private alleles multiplied by 10 of g = 12. The groupings of the mt haplotypes (west, central, east) and populations with low or no selfing versus high selfing are indicated. (PDF 1431 kb)

Additional file 5: Figure S4. Isolation By Distance (IBD) as calculated for the whole microsatellite data set; the western together with the central populations; and for the eastern population separately. (PDF 1463 kb)

Additional file 6: Table S2. Sampling site information. spId: sampling Id; IndId *COI*: individuals for which the *COI* was sequenced; IndId ms: individuals which were analyzed with microsatellites; geographic information (PDF 438 kb)

Additional file 7: Table S3. All voucher specimens were deposited in the Mollusca collection of the 3rd Zoological Department of the Natural History Museum Vienna (NHMW109000/AL/number of group/individual number). The numbers behind the last slash correspond to those in Additional file 4: Table S2. (PDF 573 kb)
