# Supplementary material for: Indication for selfing in geographically separated populations and evidence for Pleistocene survival within the Alps: the case of Cylindrus obtusus (Pulmonata: Helicidae)
Source: BMC Evol Biol. 2017 Jun 13;17:138. doi: 10.1186/s12862-017-0977-0 (PMC5470289; doi:10.1186/s12862-017-0977-0)
Supplement: Supplementary file 1 — Neighbour joining tree of the COI sequences. Each individual is defined by an individual Id, the origin of geographic region, and the Id of the sampling sites. For relevant nodes, bootstrap values are indicated. (PDF 1433 kb) [file 12862_2017_977_MOESM1_ESM.pdf]

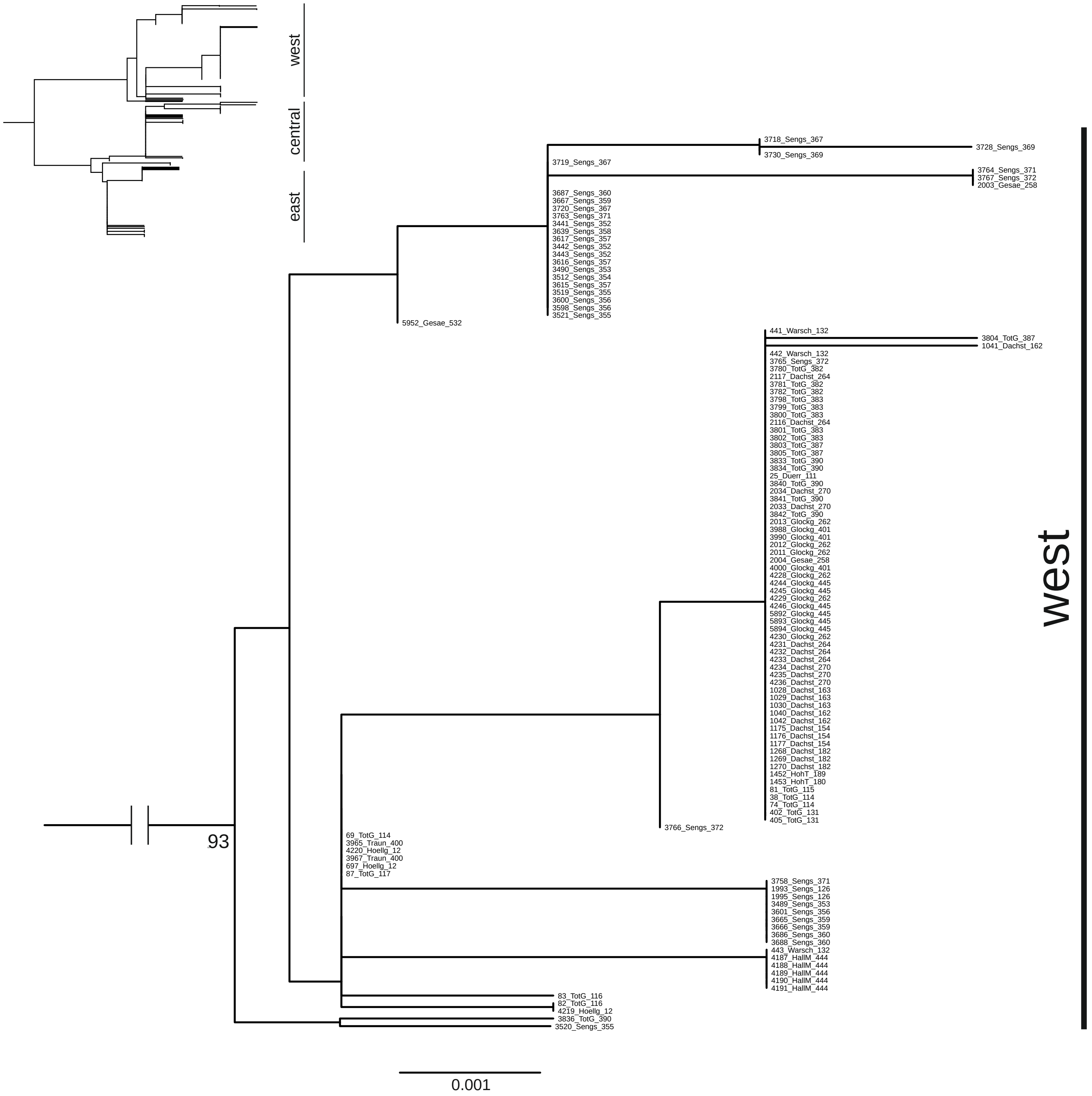

Addiəonal file 1: Figure S1. Parəal tree of the neighbour joining tree of the COI sequences illustraəng the western haplogroup in detail. Each individual is defined by an individual Id, the origin of geographic region, and the Id of the sampling sites. For relevant nodes, bootstrap values are indicated.

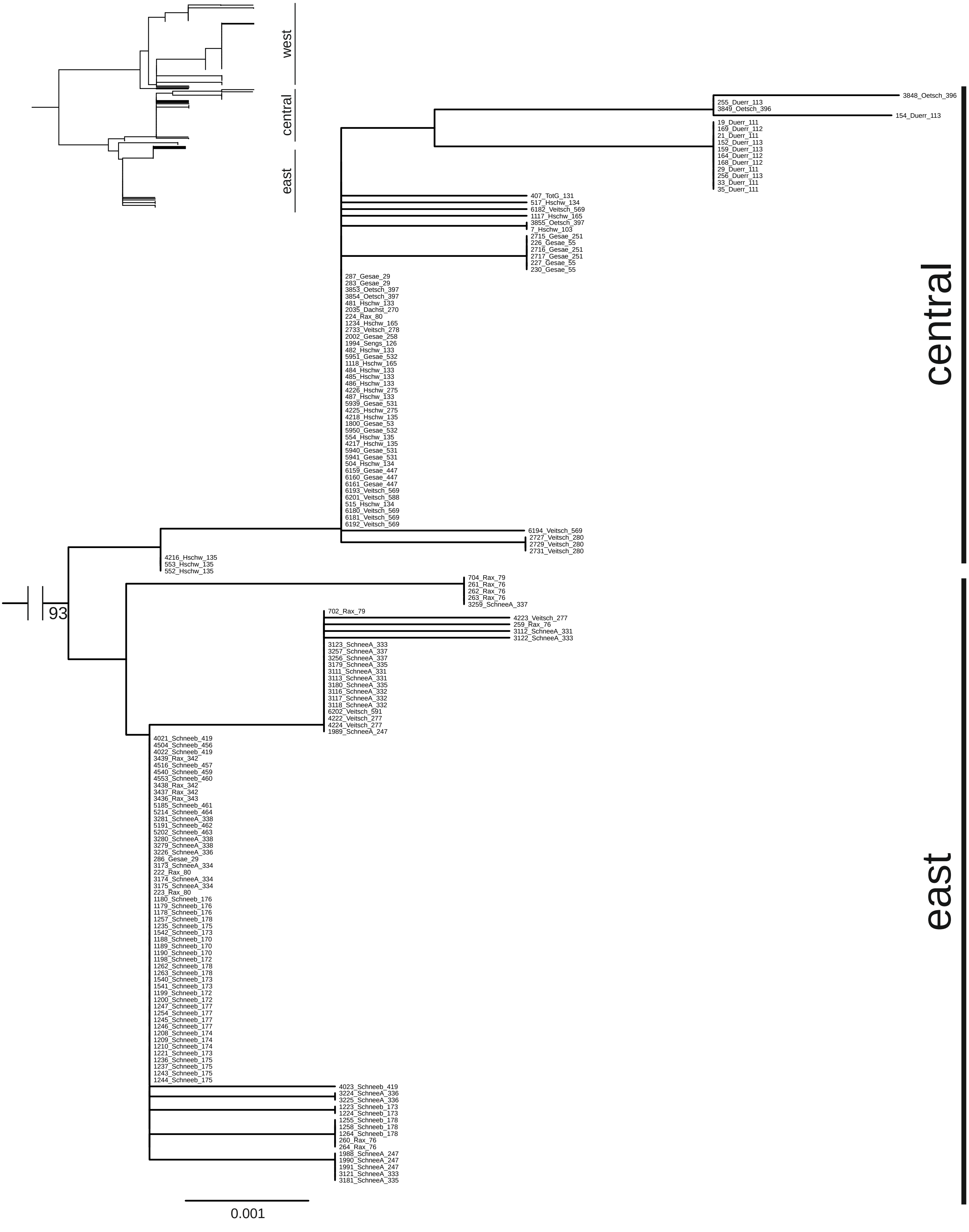

Addiəonal file 1: Figure S1. Parəal tree of the neighbour joining tree of the COI sequences illustraəng the eastern and central haplogroup in detail. Each individual is defined by an individual Id, the origin of geographic region, and the Id of the sampling sites. For relevant nodes, bootstrap values are indicated.
