# Supplementary material for: Indication for selfing in geographically separated populations and evidence for Pleistocene survival within the Alps: the case of Cylindrus obtusus (Pulmonata: Helicidae)
Source: BMC Evol Biol. 2017 Jun 13;17:138. doi: 10.1186/s12862-017-0977-0 (PMC5470289; doi:10.1186/s12862-017-0977-0)
Supplement: Supplementary file 2 — Principle component analyses (PCA) of microsatellite and sequence data. (PDF 1565 kb) [file 12862_2017_977_MOESM2_ESM.pdf]

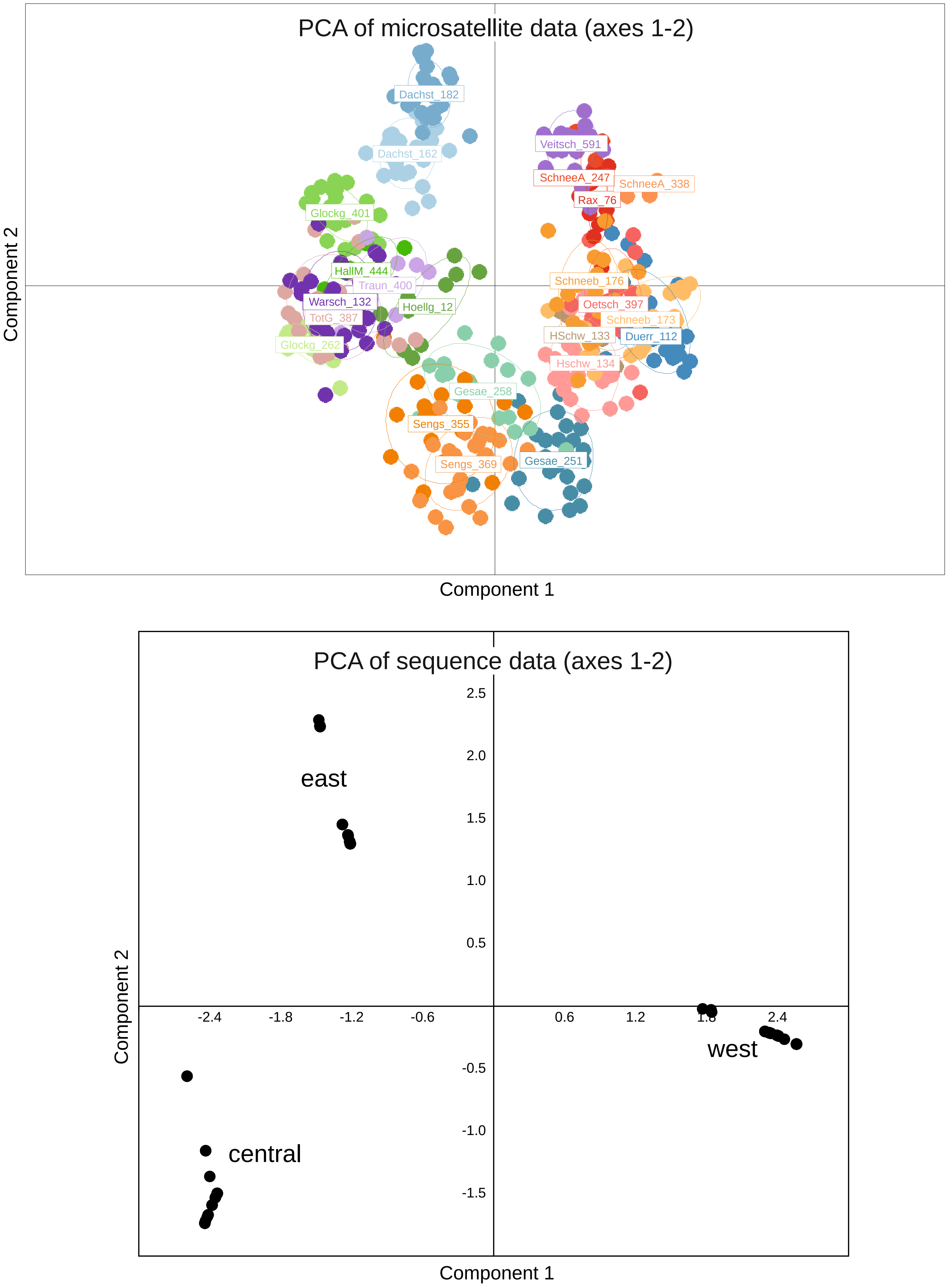

Addiǝonal file 2: Figure S2. Principle component analyses (PCA) of microsatellite and sequence data.
