# Supplementary material for: Indication for selfing in geographically separated populations and evidence for Pleistocene survival within the Alps: the case of Cylindrus obtusus (Pulmonata: Helicidae)
Source: BMC Evol Biol. 2017 Jun 13;17:138. doi: 10.1186/s12862-017-0977-0 (PMC5470289; doi:10.1186/s12862-017-0977-0)
Supplement: Supplementary file 3 — Nucleotide and haplotype diversity (standard deviation in parentheses) calculated for geographic regions. Ni = number of individuals, Nl = number of localities, H = number of haplotypes, h = haplotype diversity, pi = nucleotide diversity. (PDF 324 kb) [file 12862_2017_977_MOESM3_ESM.pdf]

Additional file 3: Table S1. Nucleotide and haplotype diversity (standard deviation in parentheses) calculated for geographic regions. Ni = number of individuals, Nl = number of localities, H = number of haplotypes, h = haplotype diversity, pi= nucleotide diversity.

Region	Abbreviation	h	pi	Ni	Nl	H
Glocknergruppe	Glockg	0	0	17	5	1
Goldberggruppe	Goldbergg	0	0	17	5	1
Dachstein	Dachst	0.1700 +/- 0.1025	0.001052 +/- 0.000921	23	6	3
Höllengebirge	Hoellg	0.6667 +/- 0.3143	0.001009 +/- 0.001258	3	1	2
Traunstein	Traun	0	0	2	1	1
Totes Gebirge	TotG	0.4558 +/- 0.1177	0.002315 +/- 0.001595	27	5	7
Warscheneck	Warsch	0.6667 +/- 0.3143	0.004034 +/- 0.003621	3	1	2
Sengsengebirge	Sengs	0.6952 +/- 0.0652	0.003446 +/- 0.002152	36	14	8
Haller Mauern	HallM	0	0	5	1	1
Gesäuse	Gesae	0.6179 +/- 0.0615	0.003572 +/- 0.002205	22	8	6
Hochschwab	Hschw	0.4857 +/- 0.1243	0.000821 +/- 0.0007	21	6	5
Dürrenstein	Duerr	0.3956 +/- 0.1588	0.003258 +/- 0.002158	14	3	4
Ötscher	Oetsch	0.9000 +/- 0.1610	0.003026 +/- 0.002376	5	2	4
Veitsch	Veitsch	0.8000 +/- 0.0771	0.002680 +/- 0.001842	15	6	6
Schneealpe	SchneeA	0.7698 +/- 0.0521	0.001881 +/- 0.001366	28	9	7
Rax	Rax	0.7905 +/- 0.0785	0.002622 +/- 0.001811	15	5	6
Schneeberg	Schneeberg	0.2526 +/- 0.0841	0.000400 +/- 0.000498	44	17	4
