# Supplementary material for: Indication for selfing in geographically separated populations and evidence for Pleistocene survival within the Alps: the case of Cylindrus obtusus (Pulmonata: Helicidae)
Source: BMC Evol Biol. 2017 Jun 13;17:138. doi: 10.1186/s12862-017-0977-0 (PMC5470289; doi:10.1186/s12862-017-0977-0)
Supplement: Supplementary file 4 — Summarising in a rarefaction approach the Allelic Richness of sample size g = 10, g = 20, and g = 30 and the mean number of private alleles multiplied by 10 of g = 12. The groupings of the mt haplotypes (west, central, east) and populations with low or no selfing versus high selfing are indicated. (PDF 1431 kb) [file 12862_2017_977_MOESM4_ESM.pdf]

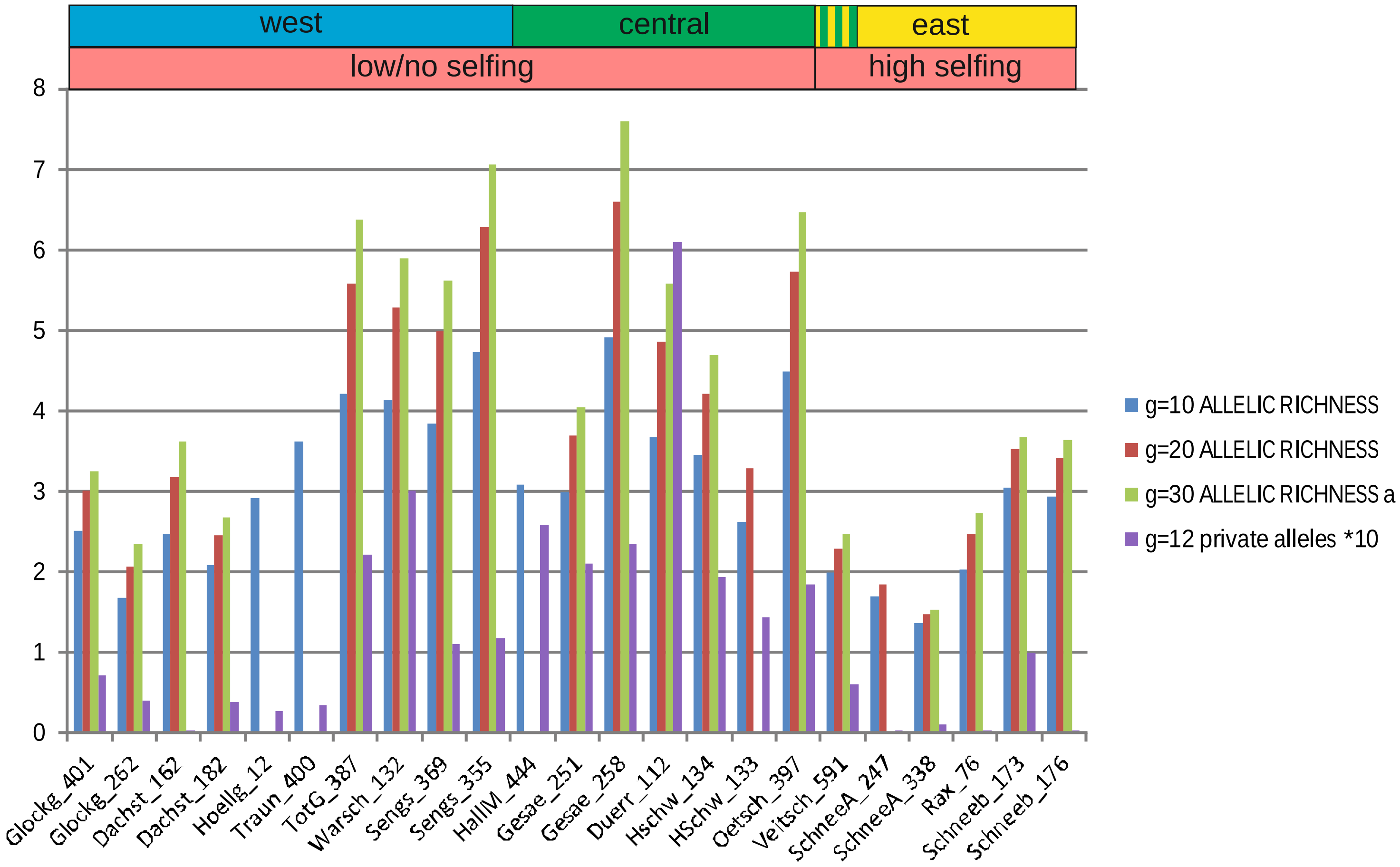

Addiǝonal file 4: Figure S3. Summarising in a rarefacǝon approach the Allelic Richness of sample size g=10, g=20, and g=30 and the mean number of private alleles mulǝplied by 10 of g=12. The groupings of the mt haplotypes (west, central, east) and populaǝons with low or no selfing versus high selfing are indicated
