# Supplementary material for: Indication for selfing in geographically separated populations and evidence for Pleistocene survival within the Alps: the case of Cylindrus obtusus (Pulmonata: Helicidae)
Source: BMC Evol Biol. 2017 Jun 13;17:138. doi: 10.1186/s12862-017-0977-0 (PMC5470289; doi:10.1186/s12862-017-0977-0)
Supplement: Supplementary file 5 — Isolation By Distance (IBD) as calculated for the whole microsatellite data set; the western together with the central populations; and for the eastern population separately. (PDF 1463 kb) [file 12862_2017_977_MOESM5_ESM.pdf]

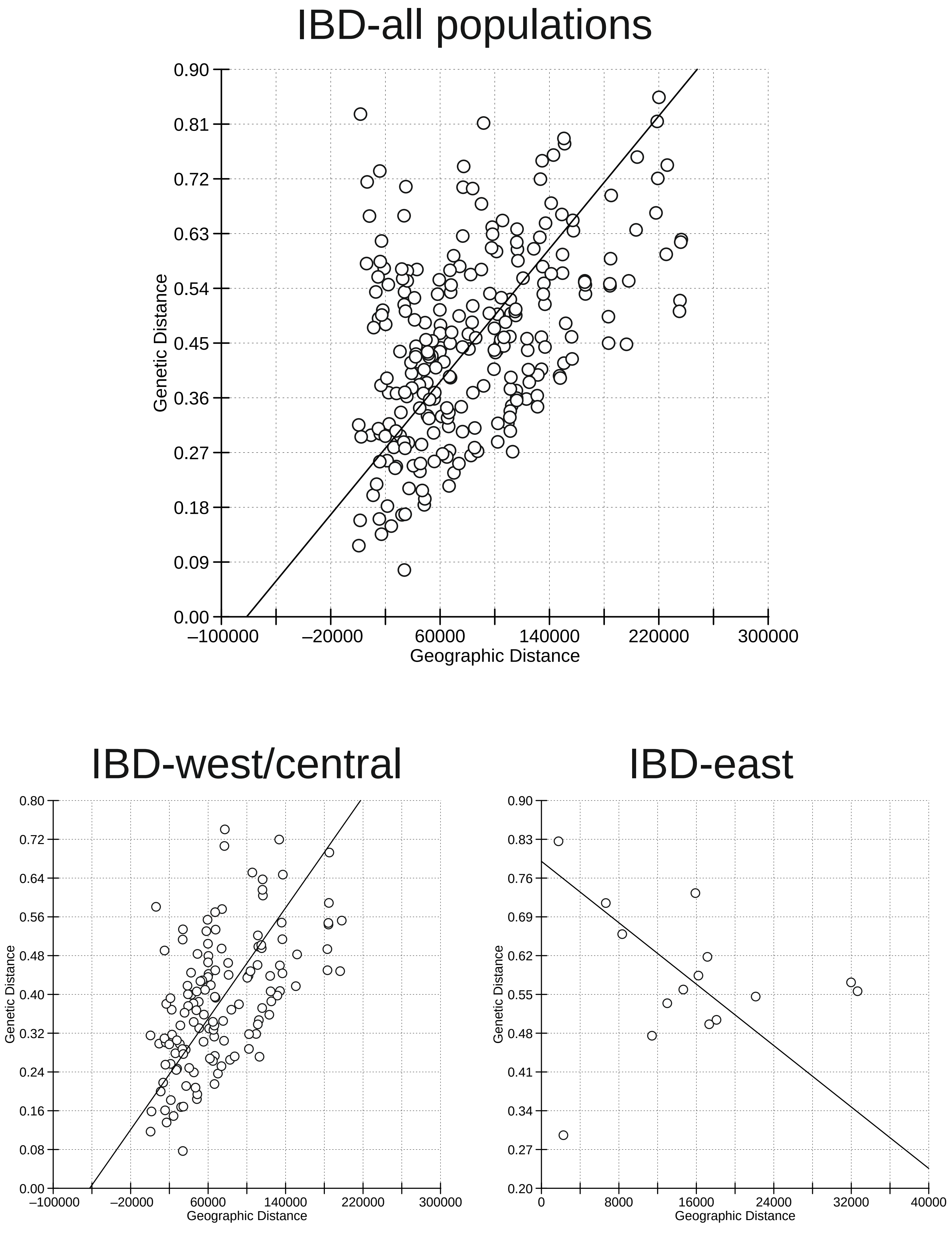

Addiəonal file 5: Figure S4. Isolaəon By Distance (IBD) as calculated for the whole microsatellite data set; the western together with the central populaəons; and for the eastern populaəons separately.
