# Supplementary material for: Indication for selfing in geographically separated populations and evidence for Pleistocene survival within the Alps: the case of Cylindrus obtusus (Pulmonata: Helicidae)
Source: BMC Evol Biol. 2017 Jun 13;17:138. doi: 10.1186/s12862-017-0977-0 (PMC5470289; doi:10.1186/s12862-017-0977-0)
Supplement: Supplementary file 6 — Sampling site information. spId: sampling Id; IndId COI: individuals for which the COI was sequenced; IndId ms: individuals which were analyzed with microsatellites; geographic information (PDF 438 kb) [file 12862_2017_977_MOESM6_ESM.pdf]

Additional file 6: Table S2. Sampling site information. spId: sampling Id; IndId COI: individuals for which the COI was sequenced; IndId ms: individuals which were analysed with microsatellites; geographic information.

spId	IndId COI	IndId ms	province	Region	latitude	longitude
12	697, 4219, 4220	694, 695, 696, 697, 698, 699, 700, 701, 4219, 4220	Upper Austria	Höllengebirge	47,82	13,71
29	283, 286, 287		Styria	Gesäuse	47,50	14,65
53	1800		Styria	Gesäuse	47,50	14,65
55	226, 227, 230		Styria	Gesäuse	47,55	14,52
76	259, 260, 261, 262, 263, 264	260, 261, 262, 263, 264, 265, 266, 267, 268, 269, 270, 271, 272, 273, 274, 276, 277, 278, 279, 281	Lower Austria	Rax	47,69	15,70
79	702, 704		Lower Austria	Rax	47,69	15,71
80	222, 223, 224		Lower Austria	Rax	47,70	15,72
103	7		Styria	Hochschwab	47,60	15,02
111	19, 21, 25, 29, 33, 35		Lower Austria	Dürrenstein	47,79	15,05
112	164, 168, 169	164, 168, 169, 171, 178, 179, 185, 186, 187, 188, 189, 190, 191, 194, 195, 197, 199, 200, 201, 203, 204, 205, 206, 209, 212, 213, 216, 217, 221	Lower Austria	Dürrenstein	47,79	15,06
113	152, 154, 159, 255, 256		Lower Austria	Dürrenstein	47,79	15,07
114	38, 69, 74		Styria	Totes Gebirge	47,68	13,96
115	81		Styria	Totes Gebirge	47,67	13,96
116	82, 83		Styria	Totes Gebirge	47,70	13,96
117	87		Styria	Totes Gebirge	47,70	13,95
126	1993, 1994, 1995		Upper Austria	Sengsengebirge	47,79	14,32
131	402, 405, 407		Styria	Totes Gebirge	47,69	13,97
132	441, 442, 443	441, 442, 443, 444, 445, 446, 447, 448, 449, 450, 451, 453, 454, 455, 456, 457, 458, 459, 460, 461, 462, 463, 465, 467, 468, 470, 471, 472, 473, 474	Upper Austria	Warscheneckgruppe	47,65	14,27
133	481, 482, 484, 485, 486, 487	481, 482, 484, 485, 486, 487, 488, 490, 491, 492	Styria	Hochschwab	47,61	15,17
134	504, 515, 517	499, 500, 501, 502, 503, 504, 507, 509, 510, 511, 512, 513, 514, 515, 517, 518, 519, 522, 525, 526, 527, 529, 533, 534, 537, 542	Styria	Hochschwab	47,62	15,15
135	552, 553, 554, 4216, 4217, 4218		Styria	Hochschwab	47,63	15,18
154	1175, 1176, 1177		Upper Austria	Dachstein	47,52	13,62

162	1040, 1041, 1042	1042, 1043, 1044, 1045, 1046, 1047, 1048, 1049, 1851, 1852, 1853, 1854, 1855, 1856, 1857, 1858, 1859, 1860, 1861, 1862, 1863, 1864, 1865, 1866, 1868, 1869, 1870, 1871, 1872	Upper Austria	Dachstein	47,52	13,62
163	1028, 1029, 1030		Upper Austria	Dachstein	47,52	13,62
165	1117, 1118, 1234		Styria	Hochschwab	47,63	15,21
170	1188, 1189, 1190		Lower Austria	Schneeberg	47,79	15,81
172	1198, 1199, 1200		Lower Austria	Schneeberg	47,79	15,81
173	1221, 1223, 1224, 1540, 1541, 1542	1218, 1219, 1220, 1221, 1222, 1223, 1224, 1225, 1226, 1227, 1530, 1531, 1532, 1533, 1534, 1535, 1536, 1537, 1538, 1539, 1540, 1541, 1542	Lower Austria	Schneeberg	47,79	15,80
174	1208, 1209, 1210		Lower Austria	Schneeberg	47,78	15,81
175	1235, 1236, 1237, 1243, 1244		Lower Austria	Schneeberg	47,78	15,81
176	1178, 1179, 1180	1178, 1179, 1180, 1181, 1182, 1183, 1184, 1185, 1186, 1187, 1900, 1901, 1902, 1903, 1904, 1905, 1906, 1907, 1908, 1909	Lower Austria	Schneeberg	47,77	15,81
177	1245, 1246, 1247, 1254		Lower Austria	Schneeberg	47,77	15,81
178	1255, 1257, 1258, 1262, 1263, 1264		Lower Austria	Schneeberg	47,76	15,83
180	1453		Carinthia	Goldberggruppe	46,97	13,03
182	1268, 1269, 1270	1268, 1269, 1270, 1271, 1272, 1273, 1274, 1275, 1276, 1277, 1839, 1840, 1841, 1842, 1843, 1844, 1845, 1846, 1847, 1848, 1849, 1850, 2757, 2758, 2759	Upper Austria	Dachstein	47,52	13,63
189	1452		Carinthia	Goldberggruppe	46,96	13,01
247	1988, 1989, 1990, 1991	1985, 1986, 1987, 1988, 1989, 1990, 1991, 2681, 2682, 2683, 2684, 2685, 2686, 2687	Styria	Schneealpe	47,70	15,59
251	2715, 2716, 2717	2700, 2701, 2702, 2703, 2704, 2705, 2706, 2707, 2708, 2709, 2710, 2711, 2712, 2713, 2714, 2715, 2716, 2717,	Styria	Gesäuse	47,55	14,52

		2718, 2719, 2720, 2721, 2722, 2723				
258	2002, 2003, 2004	2001, 2002, 2003, 2004, 2005, 2006, 2007, 2008, 2009, 2010, 2050, 2052, 2054, 2055, 2056, 2057, 2058, 2059, 2060	Styria	Gesäuse	47,62	14,69
262	2011, 2012, 2013, 4228, 4229, 4230	2011, 2012, 2013, 2014, 2015, 2016, 2017, 2018, 2019, 2020, 2021, 2022, 2023, 2024, 2025, 2026, 2027, 2028, 2029, 2030	Salzburg	Glocknergruppe	47,07	12,85
264	2116, 2117, 4231, 4232, 4233		Upper Austria	Dachstein	47,48	13,58
270	2033, 2034, 2035, 4234, 4235, 4236		Upper Austria	Dachstein	47,49	13,58
275	4225, 4226		Styria	Hochschwab	47,61	15,17
277	4222, 4223, 4224		Styria	Hohe Veitsch	47,65	15,41
278	2733		Styria	Hohe Veitsch	47,65	15,41
280	2727, 2729, 2731		Styria	Hohe Veitsch	47,65	15,41
331	3111, 3112, 3113		Styria	Schneealpe	47,70	15,60
332	3116, 3117, 3118		Styria	Schneealpe	47,70	15,60
333	3121, 3122, 3123		Styria	Schneealpe	47,70	15,60
334	3173, 3174, 3175		Styria	Schneealpe	47,70	15,60
335	3179, 3180, 3181		Styria	Schneealpe	47,70	15,60
336	3224, 3225, 3226		Styria	Schneealpe	47,70	15,59
337	3256, 3257, 3259		Styria	Schneealpe	47,70	15,61
338	3279, 3280, 3281	3279, 3280, 3281, 3282, 3283, 3284, 3285, 3286, 3287, 3288, 3289, 3290, 3291, 3292, 3293, 3294, 3295, 3296, 3424, 3425, 3426, 3427, 3428, 3429, 3430, 3431, 3432, 3433, 3434, 3435	Styria	Schneealpe	47,70	15,61
342	3437, 3438, 3439		Lower Austria	Rax	47,71	15,76
343	3436		Lower Austria	Rax	47,72	15,75
352	3441, 3442, 3443		Upper Austria	Sengsengebirge	47,79	14,32
353	3489, 3490		Upper Austria	Sengsengebirge	47,79	14,32
354	3512		Upper Austria	Sengsengebirge	47,79	14,32
355	3519, 3520, 3521	3519, 3520, 3521, 3522, 3523, 3524, 3525, 3526, 3527, 3528, 3529, 3530, 3531, 3532, 3533,	Upper Austria	Sengsengebirge	47,79	14,32

		3534, 3535, 3536, 3537				
356	3598, 3600, 3601		Upper Austria	Sengsengebirge	47,79	14,32
357	3615, 3616, 3617		Upper Austria	Sengsengebirge	47,79	14,32
358	3639		Upper Austria	Sengsengebirge	47,79	14,32
359	3665, 3666, 3667		Upper Austria	Sengsengebirge	47,79	14,32
360	3686, 3687, 3688		Upper Austria	Sengsengebirge	47,79	14,32
367	3718, 3719, 3720		Upper Austria	Sengsengebirge	47,79	14,32
369	3728, 3730	3728, 3730, 3731, 3732, 3733, 3735, 3736, 3737, 3738, 3739, 3740, 3741, 3742, 3743, 3744, 3745, 3747, 3748, 3749, 3750, 3751, 3752, 3753, 3754, 3755, 3756, 3757	Upper Austria	Sengsengebirge	47,79	14,31
371	3758, 3763, 3764		Upper Austria	Sengsengebirge	47,79	14,32
372	3765, 3766, 3767		Upper Austria	Sengsengebirge	47,79	14,32
382	3780, 3781, 3782		Upper Austria	Großer Priel	47,72	14,05
383	3798, 3799, 3800, 3801, 3802		Upper Austria	Großer Priel	47,72	14,05
387	3803, 3804, 3805	3803, 3804, 3805, 3806, 3807, 3808, 3809, 3810, 3811, 3812, 3813, 3814, 3815, 3817, 3818, 3819, 3820, 3821, 3822, 3823, 3825, 3826, 3827, 3828, 3829, 3830, 3831	Upper Austria	Großer Priel	47,71	14,05
390	3833, 3834, 3836, 3840, 3841, 3842		Upper Austria	Großer Priel	47,72	14,05
396	3848, 3849		Lower Austria	Ötscher	47,86	15,19
397	3853, 3854, 3855	3850, 3851, 3852, 3853, 3854, 3855, 3856, 3857, 3956, 3957, 3958, 3959, 3960, 3961, 3962, 3963, 3964	Lower Austria	Ötscher	47,86	15,20
400	3965, 3967	3965, 3966, 3967, 3968, 3969, 3970, 3971, 3972	Upper Austria	Traunstein	47,87	13,83
401	3988, 3990, 4000	3973, 3974, 3975, 3976, 3977, 3978, 3979, 3980, 3981, 3982, 3983, 3984, 3985, 3986, 3987, 3988, 3989, 3990, 3991, 3992, 3993, 3994, 3995, 3996, 3997, 3998, 3999, 4000	Salzburg	Glocknergruppe	47,12	12,83
419	4021, 4022, 4023		Lower Austria	Schneeberg	47,77	15,81

444	4187, 4188, 4189, 4190, 4191	4185, 4187, 4188, 4189, 4190, 4191	Styria	Haller Mauern	47,65	14,39
445	4244, 4245, 4246, 5892, 5893, 5894		Salzburg	Glocknergruppe	47,08	12,86
447	6159, 6160, 6161		Styria	Gesäuse	47,56	14,66
456	4504		Lower Austria	Schneeberg	47,77	15,81
457	4516		Lower Austria	Schneeberg	47,77	15,81
459	4540		Lower Austria	Schneeberg	47,77	15,81
460	4553		Lower Austria	Schneeberg	47,77	15,81
461	5185		Lower Austria	Schneeberg	47,77	15,81
462	5191		Lower Austria	Schneeberg	47,77	15,81
463	5202		Lower Austria	Schneeberg	47,77	15,81
464	5214		Lower Austria	Schneeberg	47,77	15,81
531	5939, 5940, 5941		Styria	Gesäuse	47,54	14,66
532	5950, 5951, 5952		Styria	Gesäuse	47,56	14,66
569	6180, 6181, 6182, 6192, 6193, 6194		Styria	Hohe Veitsch	47,66	15,41
588	6201		Styria	Hohe Veitsch	47,65	15,41
591	6202	6202, 6213, 6214, 6215, 6216, 6217, 6218, 6219, 6220, 6221, 6222, 6223, 6472, 6473, 6474, 6475, 6476, 6477, 6478, 6479, 6480, 6481, 6482, 6483, 6484	Styria	Hohe Veitsch	47,65	15,41
