# Supplementary material for: Indication for selfing in geographically separated populations and evidence for Pleistocene survival within the Alps: the case of Cylindrus obtusus (Pulmonata: Helicidae)
Source: BMC Evol Biol. 2017 Jun 13;17:138. doi: 10.1186/s12862-017-0977-0 (PMC5470289; doi:10.1186/s12862-017-0977-0)
Supplement: Supplementary file 7 — All voucher specimens were deposited in the Mollusca collection of the 3rd Zoological Department of the Natural History Museum Vienna (NHMW109000/AL/number of group/individual number). The numbers behind the last slash correspond to those in Additional file 4: Table S2. (PDF 573 kb) [file 12862_2017_977_MOESM7_ESM.pdf]

**Additional file 7: Table S3.** All voucher specimens were deposited in the collection mollusca of the 3rd Zoological Department of the Natural History Museum Vienna (NHMW109000/AL/ number of group/individual number). The numbers behind the last slash correspond to those in Additional file 6: Table S2.

| | | | |
|---|---|---|---|
| NHMW109000/AL/00001/283 | NHMW109000/AL/00001/286 | NHMW109000/AL/00001/287 | NHMW109000/AL/00003/226 |
| NHMW109000/AL/00003/227 | NHMW109000/AL/00003/230 | NHMW109000/AL/00004/7 | NHMW109000/AL/00005/38 |
| NHMW109000/AL/00005/69 | NHMW109000/AL/00005/74 | NHMW109000/AL/00006/81 | NHMW109000/AL/00007/82 |
| NHMW109000/AL/00007/83 | NHMW109000/AL/00008/87 | NHMW109000/AL/00009/402 | NHMW109000/AL/00009/405 |
| NHMW109000/AL/00009/407 | NHMW109000/AL/00010/441 | NHMW109000/AL/00010/442 | NHMW109000/AL/00010/443 |
| NHMW109000/AL/00010/444 | NHMW109000/AL/00010/445 | NHMW109000/AL/00010/446 | NHMW109000/AL/00010/447 |
| NHMW109000/AL/00010/448 | NHMW109000/AL/00010/449 | NHMW109000/AL/00010/450 | NHMW109000/AL/00010/451 |
| NHMW109000/AL/00010/453 | NHMW109000/AL/00010/454 | NHMW109000/AL/00010/455 | NHMW109000/AL/00010/456 |
| NHMW109000/AL/00010/457 | NHMW109000/AL/00010/458 | NHMW109000/AL/00010/459 | NHMW109000/AL/00010/460 |
| NHMW109000/AL/00010/461 | NHMW109000/AL/00010/462 | NHMW109000/AL/00010/463 | NHMW109000/AL/00010/465 |
| NHMW109000/AL/00010/467 | NHMW109000/AL/00010/468 | NHMW109000/AL/00010/470 | NHMW109000/AL/00010/471 |
| NHMW109000/AL/00010/472 | NHMW109000/AL/00010/473 | NHMW109000/AL/00010/474 | NHMW109000/AL/00011/1985 |
| NHMW109000/AL/00011/1986 | NHMW109000/AL/00011/1987 | NHMW109000/AL/00011/1988 | NHMW109000/AL/00011/1989 |
| NHMW109000/AL/00011/1990 | NHMW109000/AL/00011/1991 | NHMW109000/AL/00011/2681 | NHMW109000/AL/00011/2682 |
| NHMW109000/AL/00011/2683 | NHMW109000/AL/00011/2684 | NHMW109000/AL/00011/2685 | NHMW109000/AL/00011/2686 |
| NHMW109000/AL/00011/2687 | NHMW109000/AL/00012/2700 | NHMW109000/AL/00012/2701 | NHMW109000/AL/00012/2702 |
| NHMW109000/AL/00012/2703 | NHMW109000/AL/00012/2704 | NHMW109000/AL/00012/2705 | NHMW109000/AL/00012/2706 |
| NHMW109000/AL/00012/2707 | NHMW109000/AL/00012/2708 | NHMW109000/AL/00012/2709 | NHMW109000/AL/00012/2710 |
| NHMW109000/AL/00012/2711 | NHMW109000/AL/00012/2712 | NHMW109000/AL/00012/2713 | NHMW109000/AL/00012/2714 |
| NHMW109000/AL/00012/2715 | NHMW109000/AL/00012/2716 | NHMW109000/AL/00012/2717 | NHMW109000/AL/00012/2718 |
| NHMW109000/AL/00012/2719 | NHMW109000/AL/00012/2720 | NHMW109000/AL/00012/2721 | NHMW109000/AL/00012/2722 |
| NHMW109000/AL/00012/2723 | NHMW109000/AL/00013/2001 | NHMW109000/AL/00013/2002 | NHMW109000/AL/00013/2003 |
| NHMW109000/AL/00013/2004 | NHMW109000/AL/00013/2005 | NHMW109000/AL/00013/2006 | NHMW109000/AL/00013/2007 |
| NHMW109000/AL/00013/2008 | NHMW109000/AL/00013/2009 | NHMW109000/AL/00013/2010 | NHMW109000/AL/00013/2050 |
| NHMW109000/AL/00013/2052 | NHMW109000/AL/00013/2054 | NHMW109000/AL/00013/2055 | NHMW109000/AL/00013/2056 |
| NHMW109000/AL/00013/2057 | NHMW109000/AL/00013/2058 | NHMW109000/AL/00013/2059 | NHMW109000/AL/00013/2060 |
| NHMW109000/AL/00014/2011 | NHMW109000/AL/00014/2012 | NHMW109000/AL/00014/2013 | NHMW109000/AL/00014/2014 |
| NHMW109000/AL/00014/2015 | NHMW109000/AL/00014/2016 | NHMW109000/AL/00014/2017 | NHMW109000/AL/00014/2018 |
| NHMW109000/AL/00014/2019 | NHMW109000/AL/00014/2020 | NHMW109000/AL/00014/2021 | NHMW109000/AL/00014/2022 |
| NHMW109000/AL/00014/2023 | NHMW109000/AL/00014/2024 | NHMW109000/AL/00014/2025 | NHMW109000/AL/00014/2026 |
| NHMW109000/AL/00014/2027 | NHMW109000/AL/00014/2028 | NHMW109000/AL/00014/2029 | NHMW109000/AL/00014/2030 |
| NHMW109000/AL/00014/4228 | NHMW109000/AL/00014/4229 | NHMW109000/AL/00014/4230 | NHMW109000/AL/00015/2033 |
| NHMW109000/AL/00015/2034 | NHMW109000/AL/00015/2035 | NHMW109000/AL/00015/4234 | NHMW109000/AL/00015/4235 |
| NHMW109000/AL/00015/4236 | NHMW109000/AL/00016/4225 | NHMW109000/AL/00016/4226 | NHMW109000/AL/00018/4222 |
| NHMW109000/AL/00018/4223 | NHMW109000/AL/00018/4224 | NHMW109000/AL/00019/2733 | NHMW109000/AL/00021/2727 |
| NHMW109000/AL/00021/2729 | NHMW109000/AL/00021/2731 | NHMW109000/AL/00022/3111 | NHMW109000/AL/00022/3112 |
| NHMW109000/AL/00022/3113 | NHMW109000/AL/00023/3116 | NHMW109000/AL/00023/3117 | NHMW109000/AL/00023/3118 |
| NHMW109000/AL/00024/3121 | NHMW109000/AL/00024/3122 | NHMW109000/AL/00024/3123 | NHMW109000/AL/00025/3179 |
| NHMW109000/AL/00025/3180 | NHMW109000/AL/00025/3181 | NHMW109000/AL/00026/3256 | NHMW109000/AL/00026/3257 |
| NHMW109000/AL/00026/3259 | NHMW109000/AL/00027/3279 | NHMW109000/AL/00027/3280 | NHMW109000/AL/00027/3281 |
| NHMW109000/AL/00027/3282 | NHMW109000/AL/00027/3283 | NHMW109000/AL/00027/3284 | NHMW109000/AL/00027/3285 |
| NHMW109000/AL/00027/3286 | NHMW109000/AL/00027/3287 | NHMW109000/AL/00027/3288 | NHMW109000/AL/00027/3289 |
| NHMW109000/AL/00027/3290 | NHMW109000/AL/00027/3291 | NHMW109000/AL/00027/3292 | NHMW109000/AL/00027/3293 |
| NHMW109000/AL/00027/3294 | NHMW109000/AL/00027/3295 | NHMW109000/AL/00027/3296 | NHMW109000/AL/00027/3424 |
| NHMW109000/AL/00027/3425 | NHMW109000/AL/00027/3426 | NHMW109000/AL/00027/3427 | NHMW109000/AL/00027/3428 |
| NHMW109000/AL/00027/3429 | NHMW109000/AL/00027/3430 | NHMW109000/AL/00027/3431 | NHMW109000/AL/00027/3432 |
| NHMW109000/AL/00027/3433 | NHMW109000/AL/00027/3434 | NHMW109000/AL/00027/3435 | NHMW109000/AL/00028/3441 |
| NHMW109000/AL/00028/3442 | NHMW109000/AL/00028/3443 | NHMW109000/AL/00029/3489 | NHMW109000/AL/00029/3490 |
| NHMW109000/AL/00030/3512 | NHMW109000/AL/00031/3519 | NHMW109000/AL/00031/3520 | NHMW109000/AL/00031/3521 |
| NHMW109000/AL/00031/3522 | NHMW109000/AL/00031/3523 | NHMW109000/AL/00031/3524 | NHMW109000/AL/00031/3525 |
| NHMW109000/AL/00031/3526 | NHMW109000/AL/00031/3527 | NHMW109000/AL/00031/3528 | NHMW109000/AL/00031/3529 |
| NHMW109000/AL/00031/3530 | NHMW109000/AL/00031/3531 | NHMW109000/AL/00031/3532 | NHMW109000/AL/00031/3533 |
| NHMW109000/AL/00031/3534 | NHMW109000/AL/00031/3535 | NHMW109000/AL/00031/3536 | NHMW109000/AL/00031/3537 |
| NHMW109000/AL/00032/3598 | NHMW109000/AL/00032/3600 | NHMW109000/AL/00032/3601 | NHMW109000/AL/00033/3615 |
| NHMW109000/AL/00033/3616 | NHMW109000/AL/00033/3617 | NHMW109000/AL/00034/3639 | NHMW109000/AL/00035/3665 |
| NHMW109000/AL/00035/3666 | NHMW109000/AL/00035/3667 | NHMW109000/AL/00036/3686 | NHMW109000/AL/00036/3687 |
| NHMW109000/AL/00036/3688 | NHMW109000/AL/00037/3718 | NHMW109000/AL/00037/3719 | NHMW109000/AL/00037/3720 |
| NHMW109000/AL/00038/3728 | NHMW109000/AL/00038/3730 | NHMW109000/AL/00038/3731 | NHMW109000/AL/00038/3732 |
| NHMW109000/AL/00038/3733 | NHMW109000/AL/00038/3735 | NHMW109000/AL/00038/3736 | NHMW109000/AL/00038/3737 |
| NHMW109000/AL/00038/3738 | NHMW109000/AL/00038/3739 | NHMW109000/AL/00038/3740 | NHMW109000/AL/00038/3741 |

| | | | |
|---|---|---|---|
| NHMW109000/AL/00038/3742 | NHMW109000/AL/00038/3743 | NHMW109000/AL/00038/3744 | NHMW109000/AL/00038/3745 |
| NHMW109000/AL/00038/3747 | NHMW109000/AL/00038/3748 | NHMW109000/AL/00038/3749 | NHMW109000/AL/00038/3750 |
| NHMW109000/AL/00038/3751 | NHMW109000/AL/00038/3752 | NHMW109000/AL/00038/3753 | NHMW109000/AL/00038/3754 |
| NHMW109000/AL/00038/3755 | NHMW109000/AL/00038/3756 | NHMW109000/AL/00038/3757 | NHMW109000/AL/00039/3780 |
| NHMW109000/AL/00039/3781 | NHMW109000/AL/00039/3782 | NHMW109000/AL/00040/3803 | NHMW109000/AL/00040/3804 |
| NHMW109000/AL/00040/3805 | NHMW109000/AL/00040/3806 | NHMW109000/AL/00040/3807 | NHMW109000/AL/00040/3808 |
| NHMW109000/AL/00040/3809 | NHMW109000/AL/00040/3810 | NHMW109000/AL/00040/3811 | NHMW109000/AL/00040/3812 |
| NHMW109000/AL/00040/3813 | NHMW109000/AL/00040/3814 | NHMW109000/AL/00040/3815 | NHMW109000/AL/00040/3817 |
| NHMW109000/AL/00040/3818 | NHMW109000/AL/00040/3819 | NHMW109000/AL/00040/3820 | NHMW109000/AL/00040/3821 |
| NHMW109000/AL/00040/3822 | NHMW109000/AL/00040/3823 | NHMW109000/AL/00040/3825 | NHMW109000/AL/00040/3826 |
| NHMW109000/AL/00040/3827 | NHMW109000/AL/00040/3828 | NHMW109000/AL/00040/3829 | NHMW109000/AL/00040/3830 |
| NHMW109000/AL/00040/3831 | NHMW109000/AL/00041/3833 | NHMW109000/AL/00041/3834 | NHMW109000/AL/00041/3836 |
| NHMW109000/AL/00041/3840 | NHMW109000/AL/00041/3841 | NHMW109000/AL/00041/3842 | NHMW109000/AL/00042/6159 |
| NHMW109000/AL/00042/6160 | NHMW109000/AL/00042/6161 | NHMW109000/AL/00043/4504 | NHMW109000/AL/00045/4540 |
| NHMW109000/AL/00046/4553 | NHMW109000/AL/00047/5185 | NHMW109000/AL/00048/5191 | NHMW109000/AL/00049/5202 |
| NHMW109000/AL/00050/5214 | NHMW109000/AL/00052/1800 | NHMW109000/AL/00054/1198 | NHMW109000/AL/00054/1199 |
| NHMW109000/AL/00054/1200 | NHMW109000/AL/00055/1245 | NHMW109000/AL/00055/1246 | NHMW109000/AL/00055/1247 |
| NHMW109000/AL/00055/1254 | NHMW109000/AL/00056/3224 | NHMW109000/AL/00056/3225 | NHMW109000/AL/00056/3226 |
| NHMW109000/AL/00057/3437 | NHMW109000/AL/00057/3438 | NHMW109000/AL/00057/3439 | NHMW109000/AL/00058/3436 |
| NHMW109000/AL/00059/3848 | NHMW109000/AL/00059/3849 | NHMW109000/AL/00060/3850 | NHMW109000/AL/00060/3851 |
| NHMW109000/AL/00060/3852 | NHMW109000/AL/00060/3853 | NHMW109000/AL/00060/3854 | NHMW109000/AL/00060/3855 |
| NHMW109000/AL/00060/3856 | NHMW109000/AL/00060/3857 | NHMW109000/AL/00060/3956 | NHMW109000/AL/00060/3957 |
| NHMW109000/AL/00060/3958 | NHMW109000/AL/00060/3959 | NHMW109000/AL/00060/3960 | NHMW109000/AL/00060/3961 |
| NHMW109000/AL/00060/3962 | NHMW109000/AL/00060/3963 | NHMW109000/AL/00060/3964 | NHMW109000/AL/00061/4021 |
| NHMW109000/AL/00061/4022 | NHMW109000/AL/00061/4023 | NHMW109000/AL/00062/4185 | NHMW109000/AL/00062/4187 |
| NHMW109000/AL/00062/4188 | NHMW109000/AL/00062/4189 | NHMW109000/AL/00062/4190 | NHMW109000/AL/00062/4191 |
| NHMW109000/AL/00063/4244 | NHMW109000/AL/00063/4245 | NHMW109000/AL/00063/4246 | NHMW109000/AL/00063/5892 |
| NHMW109000/AL/00063/5893 | NHMW109000/AL/00063/5894 | NHMW109000/AL/00065/5939 | NHMW109000/AL/00065/5940 |
| NHMW109000/AL/00065/5941 | NHMW109000/AL/00066/5950 | NHMW109000/AL/00066/5951 | NHMW109000/AL/00066/5952 |
| NHMW109000/AL/00067/6202 | NHMW109000/AL/00067/6213 | NHMW109000/AL/00067/6214 | NHMW109000/AL/00067/6215 |
| NHMW109000/AL/00067/6216 | NHMW109000/AL/00067/6217 | NHMW109000/AL/00067/6218 | NHMW109000/AL/00067/6219 |
| NHMW109000/AL/00067/6220 | NHMW109000/AL/00067/6221 | NHMW109000/AL/00067/6222 | NHMW109000/AL/00067/6223 |
| NHMW109000/AL/00067/6472 | NHMW109000/AL/00067/6473 | NHMW109000/AL/00067/6474 | NHMW109000/AL/00067/6475 |
| NHMW109000/AL/00067/6476 | NHMW109000/AL/00067/6477 | NHMW109000/AL/00067/6478 | NHMW109000/AL/00067/6479 |
| NHMW109000/AL/00067/6480 | NHMW109000/AL/00067/6481 | NHMW109000/AL/00067/6482 | NHMW109000/AL/00067/6483 |
| NHMW109000/AL/00067/6484 | NHMW109000/AL/00074/1028 | NHMW109000/AL/00074/1029 | NHMW109000/AL/00074/1030 |
| NHMW109000/AL/00347/4219 | NHMW109000/AL/00347/4220 | NHMW109000/AL/00347/694 | NHMW109000/AL/00347/695 |
| NHMW109000/AL/00347/696 | NHMW109000/AL/00347/697 | NHMW109000/AL/00347/698 | NHMW109000/AL/00347/699 |
| NHMW109000/AL/00347/700 | NHMW109000/AL/00347/701 | NHMW109000/AL/00348/259 | NHMW109000/AL/00348/260 |
| NHMW109000/AL/00348/261 | NHMW109000/AL/00348/262 | NHMW109000/AL/00348/263 | NHMW109000/AL/00348/264 |
| NHMW109000/AL/00348/265 | NHMW109000/AL/00348/266 | NHMW109000/AL/00348/267 | NHMW109000/AL/00348/268 |
| NHMW109000/AL/00348/269 | NHMW109000/AL/00348/270 | NHMW109000/AL/00348/271 | NHMW109000/AL/00348/272 |
| NHMW109000/AL/00348/273 | NHMW109000/AL/00348/274 | NHMW109000/AL/00348/276 | NHMW109000/AL/00348/277 |
| NHMW109000/AL/00348/278 | NHMW109000/AL/00348/279 | NHMW109000/AL/00348/281 | NHMW109000/AL/00349/702 |
| NHMW109000/AL/00349/704 | NHMW109000/AL/00350/222 | NHMW109000/AL/00350/223 | NHMW109000/AL/00350/224 |
| NHMW109000/AL/00351/19 | NHMW109000/AL/00351/21 | NHMW109000/AL/00351/25 | NHMW109000/AL/00351/29 |
| NHMW109000/AL/00351/33 | NHMW109000/AL/00351/35 | NHMW109000/AL/00352/164 | NHMW109000/AL/00352/168 |
| NHMW109000/AL/00352/169 | NHMW109000/AL/00352/171 | NHMW109000/AL/00352/178 | NHMW109000/AL/00352/179 |
| NHMW109000/AL/00352/185 | NHMW109000/AL/00352/186 | NHMW109000/AL/00352/187 | NHMW109000/AL/00352/188 |
| NHMW109000/AL/00352/189 | NHMW109000/AL/00352/190 | NHMW109000/AL/00352/191 | NHMW109000/AL/00352/194 |
| NHMW109000/AL/00352/195 | NHMW109000/AL/00352/197 | NHMW109000/AL/00352/199 | NHMW109000/AL/00352/200 |
| NHMW109000/AL/00352/201 | NHMW109000/AL/00352/203 | NHMW109000/AL/00352/204 | NHMW109000/AL/00352/205 |
| NHMW109000/AL/00352/206 | NHMW109000/AL/00352/209 | NHMW109000/AL/00352/212 | NHMW109000/AL/00352/213 |
| NHMW109000/AL/00352/216 | NHMW109000/AL/00352/217 | NHMW109000/AL/00352/221 | NHMW109000/AL/00353/152 |
| NHMW109000/AL/00353/154 | NHMW109000/AL/00353/159 | NHMW109000/AL/00353/255 | NHMW109000/AL/00353/256 |
| NHMW109000/AL/00354/1993 | NHMW109000/AL/00354/1994 | NHMW109000/AL/00354/1995 | NHMW109000/AL/00355/481 |
| NHMW109000/AL/00355/482 | NHMW109000/AL/00355/484 | NHMW109000/AL/00355/485 | NHMW109000/AL/00355/486 |
| NHMW109000/AL/00355/487 | NHMW109000/AL/00355/488 | NHMW109000/AL/00355/490 | NHMW109000/AL/00355/491 |
| NHMW109000/AL/00355/492 | NHMW109000/AL/00356/499 | NHMW109000/AL/00356/500 | NHMW109000/AL/00356/501 |
| NHMW109000/AL/00356/502 | NHMW109000/AL/00356/503 | NHMW109000/AL/00356/504 | NHMW109000/AL/00356/507 |
| NHMW109000/AL/00356/509 | NHMW109000/AL/00356/510 | NHMW109000/AL/00356/511 | NHMW109000/AL/00356/512 |
| NHMW109000/AL/00356/513 | NHMW109000/AL/00356/514 | NHMW109000/AL/00356/515 | NHMW109000/AL/00356/517 |
| NHMW109000/AL/00356/518 | NHMW109000/AL/00356/519 | NHMW109000/AL/00356/522 | NHMW109000/AL/00356/525 |
| NHMW109000/AL/00356/526 | NHMW109000/AL/00356/527 | NHMW109000/AL/00356/529 | NHMW109000/AL/00356/533 |
| NHMW109000/AL/00356/534 | NHMW109000/AL/00356/537 | NHMW109000/AL/00356/542 | NHMW109000/AL/00357/4216 |
| NHMW109000/AL/00357/4217 | NHMW109000/AL/00357/4218 | NHMW109000/AL/00357/552 | NHMW109000/AL/00357/553 |
| NHMW109000/AL/00357/554 | NHMW109000/AL/00358/1175 | NHMW109000/AL/00358/1176 | NHMW109000/AL/00358/1177 |
| NHMW109000/AL/00359/1040 | NHMW109000/AL/00359/1041 | NHMW109000/AL/00359/1042 | NHMW109000/AL/00359/1043 |
| NHMW109000/AL/00359/1044 | NHMW109000/AL/00359/1045 | NHMW109000/AL/00359/1046 | NHMW109000/AL/00359/1047 |

| | | | |
|---|---|---|---|
| NHMW109000/AL/00359/1048 | NHMW109000/AL/00359/1049 | NHMW109000/AL/00359/1851 | NHMW109000/AL/00359/1852 |
| NHMW109000/AL/00359/1853 | NHMW109000/AL/00359/1854 | NHMW109000/AL/00359/1855 | NHMW109000/AL/00359/1856 |
| NHMW109000/AL/00359/1857 | NHMW109000/AL/00359/1858 | NHMW109000/AL/00359/1859 | NHMW109000/AL/00359/1860 |
| NHMW109000/AL/00359/1861 | NHMW109000/AL/00359/1862 | NHMW109000/AL/00359/1863 | NHMW109000/AL/00359/1864 |
| NHMW109000/AL/00359/1865 | NHMW109000/AL/00359/1866 | NHMW109000/AL/00359/1868 | NHMW109000/AL/00359/1869 |
| NHMW109000/AL/00359/1870 | NHMW109000/AL/00359/1871 | NHMW109000/AL/00359/1872 | NHMW109000/AL/00360/1117 |
| NHMW109000/AL/00360/1118 | NHMW109000/AL/00360/1234 | NHMW109000/AL/00361/1188 | NHMW109000/AL/00361/1189 |
| NHMW109000/AL/00361/1190 | NHMW109000/AL/00362/1218 | NHMW109000/AL/00362/1219 | NHMW109000/AL/00362/1220 |
| NHMW109000/AL/00362/1221 | NHMW109000/AL/00362/1222 | NHMW109000/AL/00362/1223 | NHMW109000/AL/00362/1224 |
| NHMW109000/AL/00362/1225 | NHMW109000/AL/00362/1226 | NHMW109000/AL/00362/1227 | NHMW109000/AL/00362/1530 |
| NHMW109000/AL/00362/1531 | NHMW109000/AL/00362/1532 | NHMW109000/AL/00362/1533 | NHMW109000/AL/00362/1534 |
| NHMW109000/AL/00362/1535 | NHMW109000/AL/00362/1536 | NHMW109000/AL/00362/1537 | NHMW109000/AL/00362/1538 |
| NHMW109000/AL/00362/1539 | NHMW109000/AL/00362/1540 | NHMW109000/AL/00362/1541 | NHMW109000/AL/00362/1542 |
| NHMW109000/AL/00363/1235 | NHMW109000/AL/00363/1236 | NHMW109000/AL/00363/1237 | NHMW109000/AL/00363/1243 |
| NHMW109000/AL/00363/1244 | NHMW109000/AL/00364/1178 | NHMW109000/AL/00364/1179 | NHMW109000/AL/00364/1180 |
| NHMW109000/AL/00364/1181 | NHMW109000/AL/00364/1182 | NHMW109000/AL/00364/1183 | NHMW109000/AL/00364/1184 |
| NHMW109000/AL/00364/1185 | NHMW109000/AL/00364/1186 | NHMW109000/AL/00364/1187 | NHMW109000/AL/00364/1900 |
| NHMW109000/AL/00364/1901 | NHMW109000/AL/00364/1902 | NHMW109000/AL/00364/1903 | NHMW109000/AL/00364/1904 |
| NHMW109000/AL/00364/1905 | NHMW109000/AL/00364/1906 | NHMW109000/AL/00364/1907 | NHMW109000/AL/00364/1908 |
| NHMW109000/AL/00364/1909 | NHMW109000/AL/00365/1255 | NHMW109000/AL/00365/1257 | NHMW109000/AL/00365/1258 |
| NHMW109000/AL/00365/1262 | NHMW109000/AL/00365/1263 | NHMW109000/AL/00365/1264 | NHMW109000/AL/00366/1453 |
| NHMW109000/AL/00367/1268 | NHMW109000/AL/00367/1269 | NHMW109000/AL/00367/1270 | NHMW109000/AL/00367/1271 |
| NHMW109000/AL/00367/1272 | NHMW109000/AL/00367/1273 | NHMW109000/AL/00367/1274 | NHMW109000/AL/00367/1275 |
| NHMW109000/AL/00367/1276 | NHMW109000/AL/00367/1277 | NHMW109000/AL/00367/1839 | NHMW109000/AL/00367/1840 |
| NHMW109000/AL/00367/1841 | NHMW109000/AL/00367/1842 | NHMW109000/AL/00367/1843 | NHMW109000/AL/00367/1844 |
| NHMW109000/AL/00367/1845 | NHMW109000/AL/00367/1846 | NHMW109000/AL/00367/1847 | NHMW109000/AL/00367/1848 |
| NHMW109000/AL/00367/1849 | NHMW109000/AL/00367/1850 | NHMW109000/AL/00367/2757 | NHMW109000/AL/00367/2758 |
| NHMW109000/AL/00367/2759 | NHMW109000/AL/00368/1452 | NHMW109000/AL/00369/2116 | NHMW109000/AL/00369/2117 |
| NHMW109000/AL/00369/4231 | NHMW109000/AL/00369/4232 | NHMW109000/AL/00369/4233 | NHMW109000/AL/00370/3173 |
| NHMW109000/AL/00370/3174 | NHMW109000/AL/00370/3175 | NHMW109000/AL/00371/3758 | NHMW109000/AL/00371/3763 |
| NHMW109000/AL/00371/3764 | NHMW109000/AL/00372/3765 | NHMW109000/AL/00372/3766 | NHMW109000/AL/00372/3767 |
| NHMW109000/AL/00373/3798 | NHMW109000/AL/00373/3799 | NHMW109000/AL/00373/3800 | NHMW109000/AL/00373/3801 |
| NHMW109000/AL/00373/3802 | NHMW109000/AL/00374/3965 | NHMW109000/AL/00374/3966 | NHMW109000/AL/00374/3967 |
| NHMW109000/AL/00374/3968 | NHMW109000/AL/00374/3969 | NHMW109000/AL/00374/3970 | NHMW109000/AL/00374/3971 |
| NHMW109000/AL/00374/3972 | NHMW109000/AL/00375/3973 | NHMW109000/AL/00375/3974 | NHMW109000/AL/00375/3975 |
| NHMW109000/AL/00375/3976 | NHMW109000/AL/00375/3977 | NHMW109000/AL/00375/3978 | NHMW109000/AL/00375/3979 |
| NHMW109000/AL/00375/3980 | NHMW109000/AL/00375/3981 | NHMW109000/AL/00375/3982 | NHMW109000/AL/00375/3983 |
| NHMW109000/AL/00375/3984 | NHMW109000/AL/00375/3985 | NHMW109000/AL/00375/3986 | NHMW109000/AL/00375/3987 |
| NHMW109000/AL/00375/3988 | NHMW109000/AL/00375/3989 | NHMW109000/AL/00375/3990 | NHMW109000/AL/00375/3991 |
| NHMW109000/AL/00375/3992 | NHMW109000/AL/00375/3993 | NHMW109000/AL/00375/3994 | NHMW109000/AL/00375/3995 |
| NHMW109000/AL/00375/3996 | NHMW109000/AL/00375/3997 | NHMW109000/AL/00375/3998 | NHMW109000/AL/00375/3999 |
| NHMW109000/AL/00375/4000 | NHMW109000/AL/00376/4516 | NHMW109000/AL/00377/6180 | NHMW109000/AL/00377/6181 |
| NHMW109000/AL/00377/6182 | NHMW109000/AL/00377/6192 | NHMW109000/AL/00377/6193 | NHMW109000/AL/00377/6194 |
| NHMW109000/AL/00378/6201 | NHMW109000/AL/00479/1208 | NHMW109000/AL/00479/1209 | NHMW109000/AL/00479/1210 |
